# Supplementary figures and images for: PIP2 regulating calcium signal modulates actin cytoskeleton-dependent cytoadherence and cytolytic capacity in the protozoan parasite Trichomonas vaginalis
Source: PLoS Pathog. 2023 Dec 18;19(12):e1011891. doi: 10.1371/journal.ppat.1011891 (PMC10758264; doi:10.1371/journal.ppat.1011891)

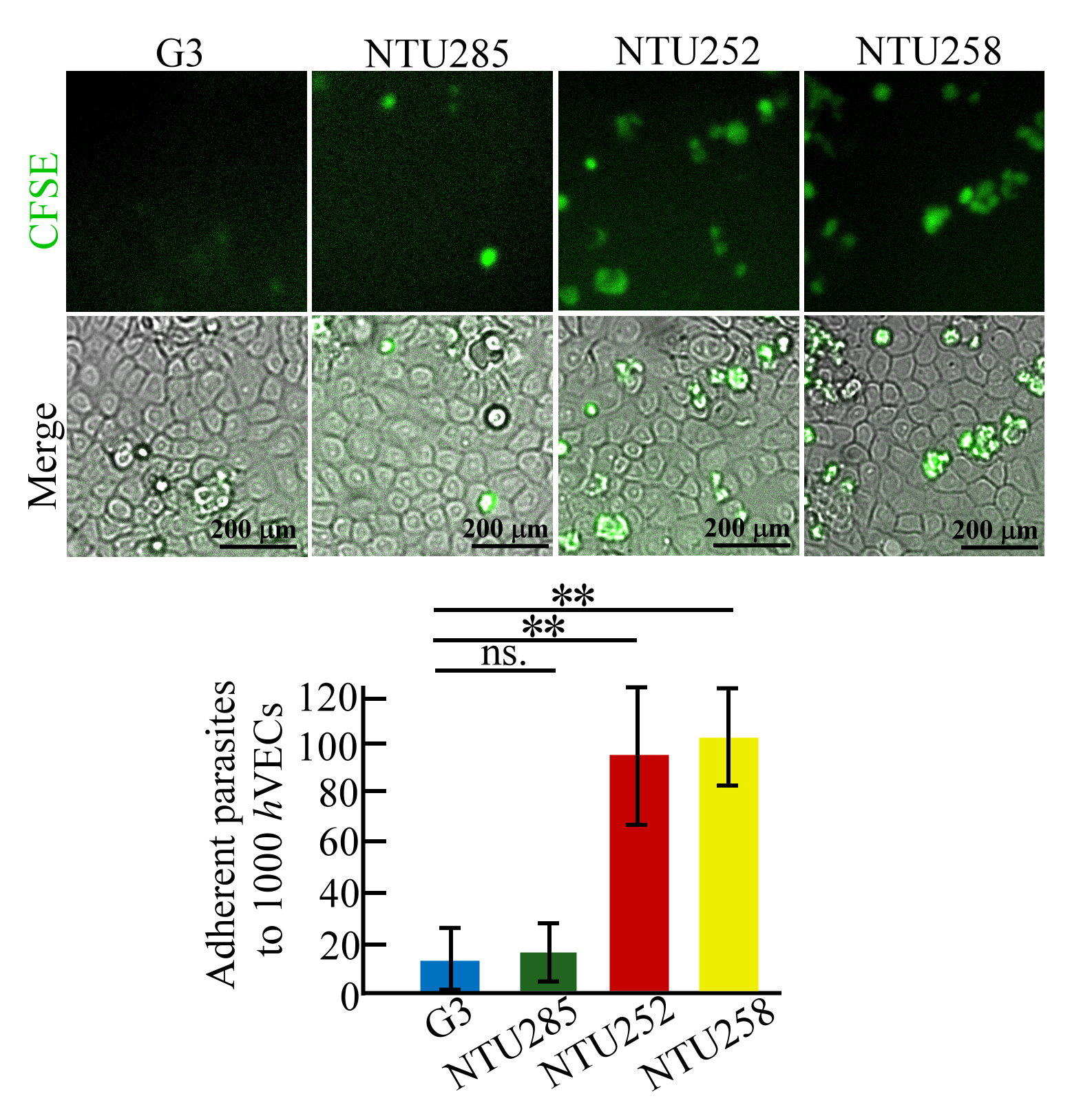

Supplement: S1 Fig — The CFSE-labeled trophozoites from G3 and NTU252, NTU258, and NTU285 clinical isolates, were co-cultured with hVECs for cytoadherence assay. The CFSE signal was recorded by fluorescence microscopy and quantified by calculating the number of adherent parasites to 1000 hVECs. The assay was processed in three biological repeats (n = 3, mean ± SD). The significant differences for the paired samples were analyzed by Student’s t-tests, with p< 0.05(*), p< 0.01(**), ns. no significant difference. (TIF) [file ppat.1011891.s001.tif]

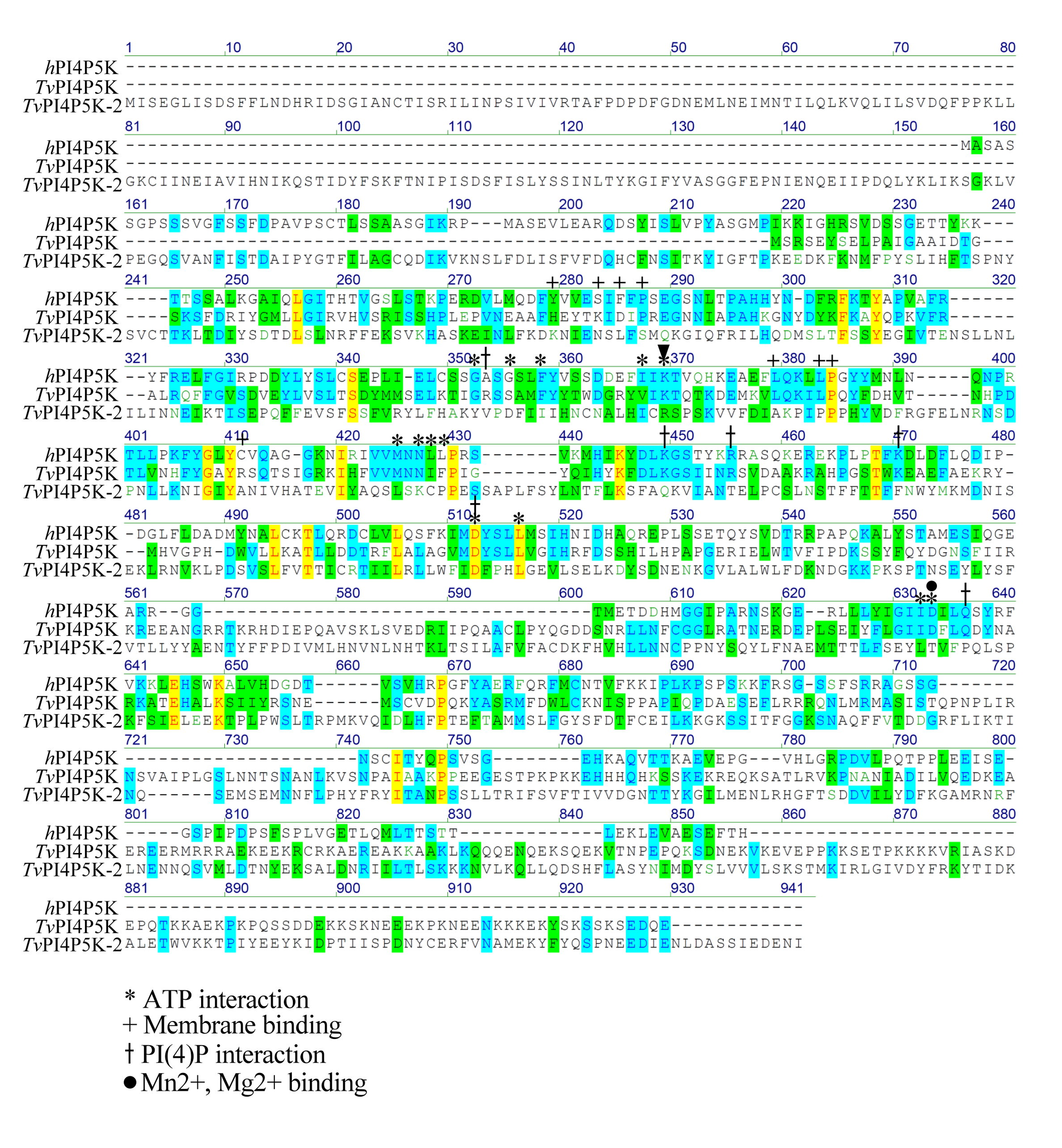

Supplement: S2 Fig — The alignment of human hPI4P5K (Q99755), T. vaginalis TvPI4P5K (TVAG_462290), and TvPI4P5K-2 (TVAG_456620), with identical or similar amino acids highlighted. The putative sites for ATP interaction (*), membrane binding (+), PI4P substrate binding (†), Mn2+ and Mg2+ binding (⬤), and the kinase-activity-essential K136 residue (▼) are indicated at the top of sequences. (TIF) [file ppat.1011891.s002.tif]

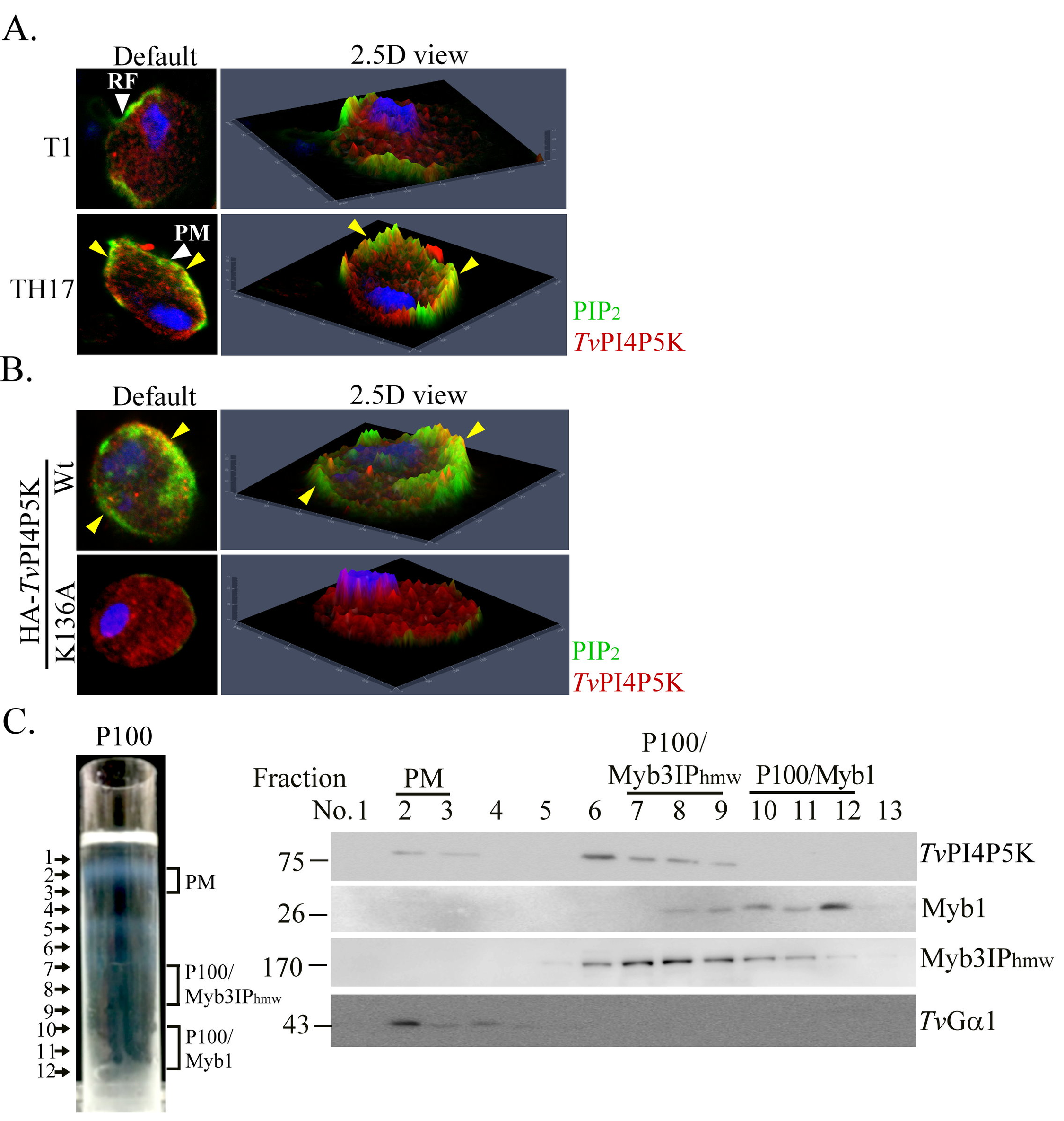

Supplement: S3 Fig — The IFA images from Fig 2E and 2I were viewed by 2.5D view of ZEN software as shown in (A) and (B), respectively. The intensities in the two-dimensional image were converted into a height map and are represented by the extension in the Z-direction. RF indicates recurrent flagellum and PM indicates plasma membrane. The yellow arrowheads indicate colocalization (yellow) of TvPI4P5K (red) and PIP2 (green) in particular plasma membrane regions. (C) P100 was fractionated by OptiPrep density gradient ultracentrifugation (left panel) and 200-μl aliquots were collected from the top of each gradient for western blotting using antibodies as indicated. Myb1, Myb3IPhmw, and TvGα protein were detected as membrane compartment markers for P100/Myb1, P100/Myb3IPhmw, and the plasma membrane (PM), respectively. (TIF) [file ppat.1011891.s003.tif]

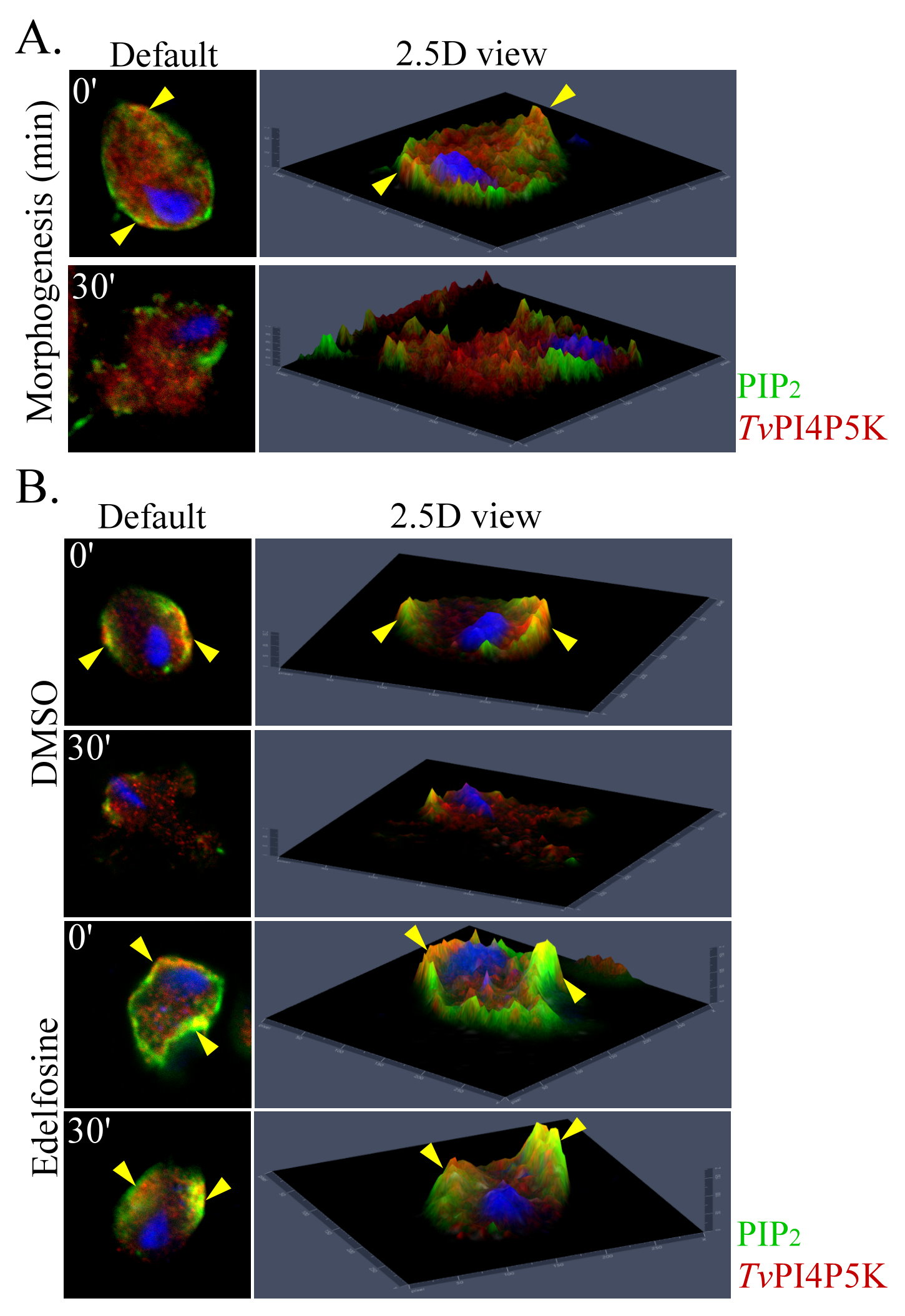

Supplement: S4 Fig — The IFA images from Fig 3D and 3E were viewed by 2.5D view of ZEN software as shown in (A) and (B), respectively. The intensities in a two-dimensional image were converted into a height map and represented by the extension in the Z-direction. The yellow arrowheads indicate colocalization (yellow) of TvPI4P5K (red) and PIP2 (green) at specific plasma membrane regions. (TIF) [file ppat.1011891.s004.tif]

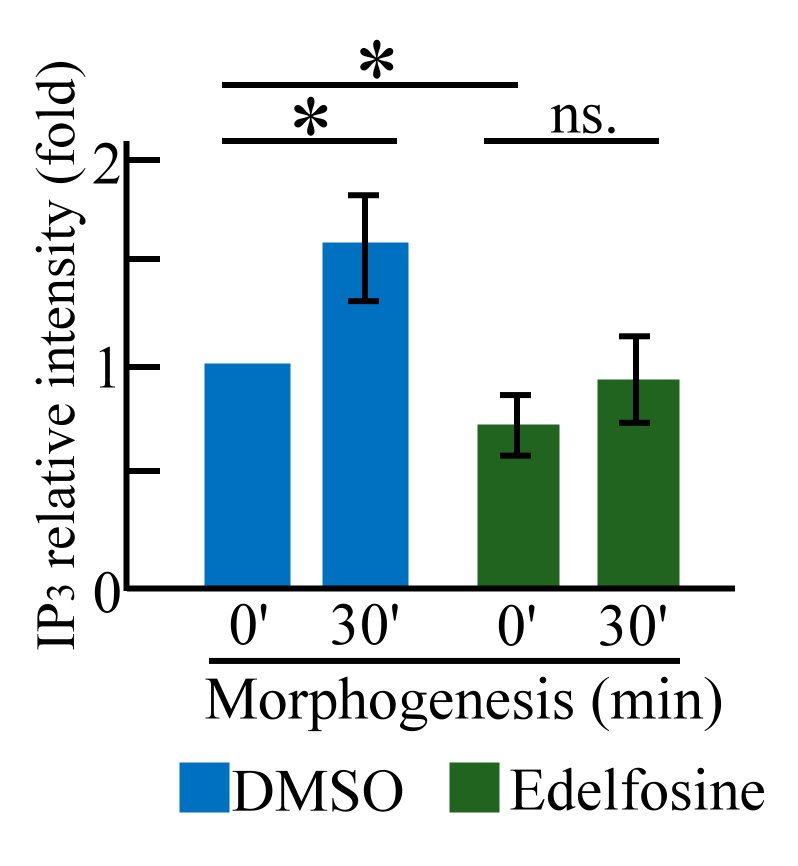

Supplement: S5 Fig — The cell lysates from trophozoites before (0′) and after amoeboid transition (30′) were detected with a commercial IP3 ELISA kit, and the relative colorimetric signal was analyzed by a spectrophotometer at OD450. The assay was processed in three biological repeats (n = 3, mean ± SD). The significant differences for the paired samples were analyzed by Student’s t-tests, with p< 0.05(*), p< 0.01(**), ns. no significant difference. (TIF) [file ppat.1011891.s005.tif]

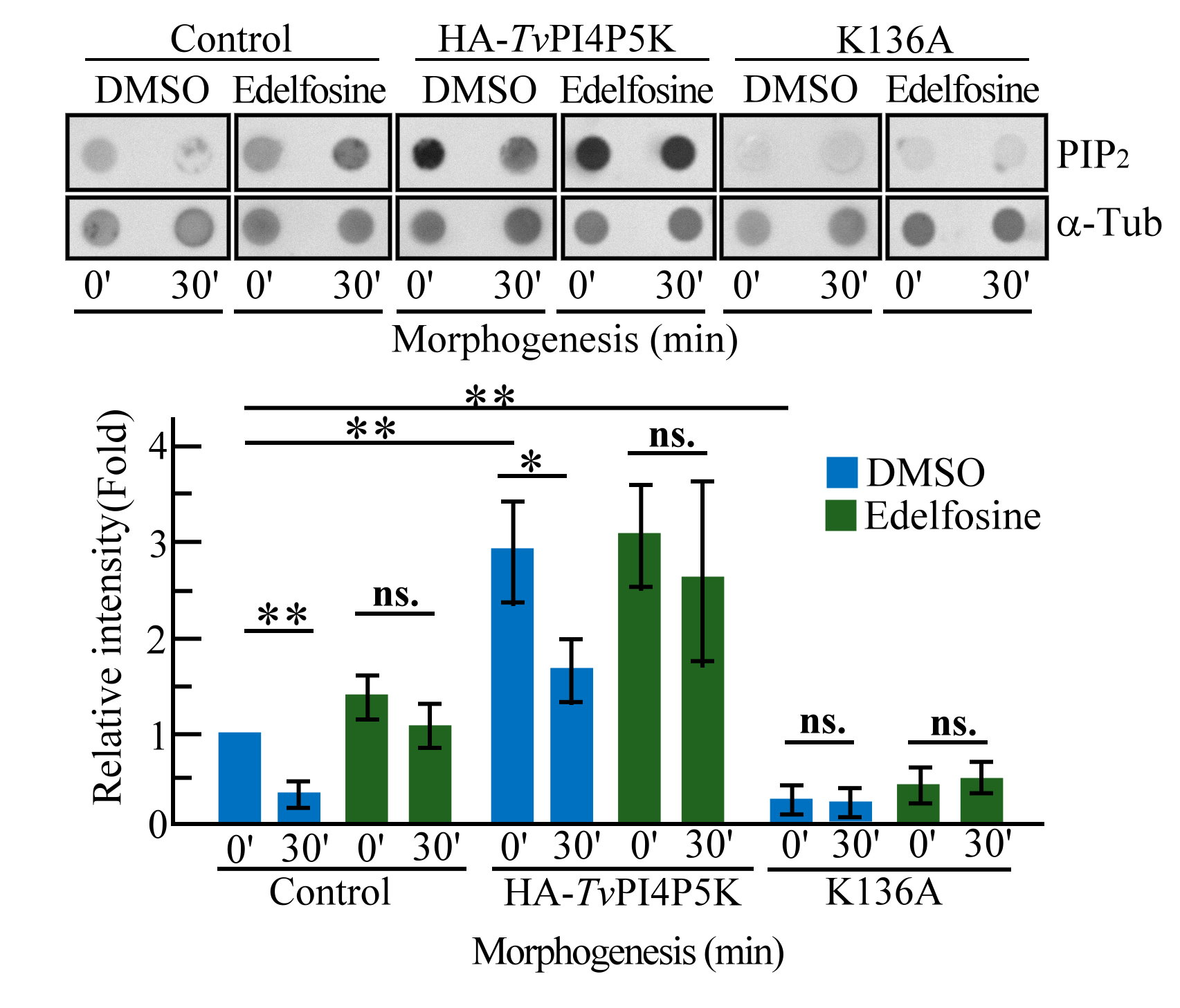

Supplement: S6 Fig — The protein lysates extracted from the non-transgenic and transgenic trophozoites with or without Edelfosine treatment before (0′) and after culture in a T25 flask for 30 min (30′) were subjected to dot blot for PIP2 and α-tubulin detection. The assay was processed in three biological repeats, and the relative PIP2 signal intensity normalized to α-tubulin is shown in the bar graph (n = 3, mean ± SD). The significant differences for the paired conditional samples were analyzed by Student’s t-tests, with p< 0.05(*), p< 0.01(**), ns. no significant difference. (TIF) [file ppat.1011891.s006.tif]

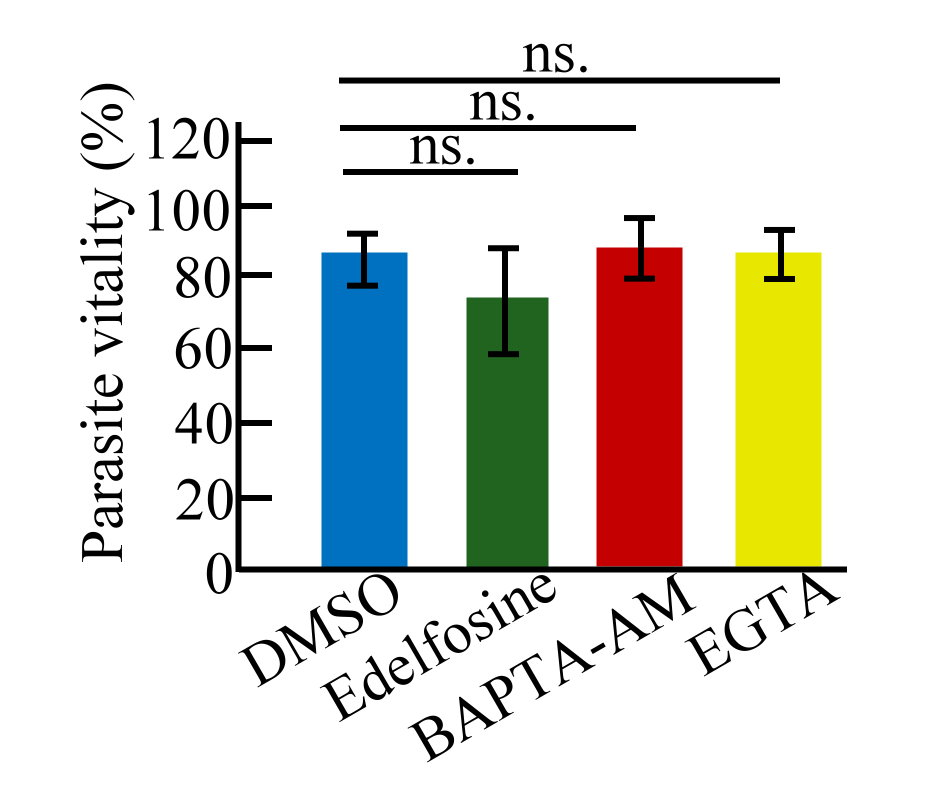

Supplement: S7 Fig — The viability of T. vaginalis trophozoites treated with DMSO, Edelfosine, BAPTA-AM, or EGTA was analyzed using the Trypan blue exclusion assay. The assay was processed in three biological repeats (n = 3, mean ± SD). The significant differences for the paired samples were analyzed by Student’s t-tests, with p< 0.05(*), p< 0.01(**), ns. no significant difference. (TIF) [file ppat.1011891.s007.tif]

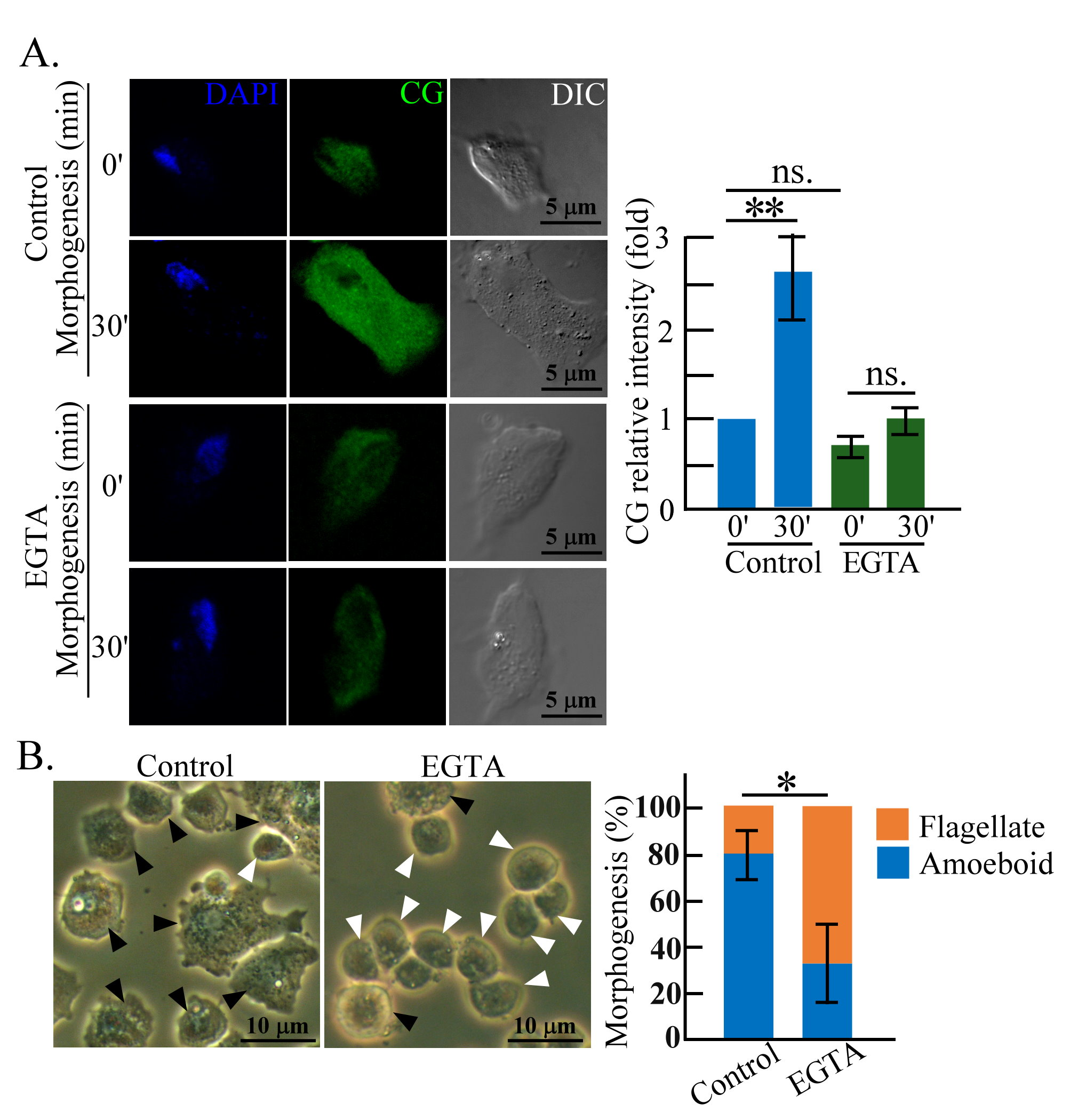

Supplement: S8 Fig — The CG-preloaded TH17 flagellates were inoculated in the medium with or without EGTA and incubated on a glass slide for 30 min. The parasites before (0′) and after (30′) morphogenesis were fixed for CG detection (A) or morphogenesis assay (B). (A) The relative CG signal intensity was quantified as shown in the bar graph. (B) The percentage of flagellate versus amoeboid trophozoites was measured as shown in the bar graph. Black and white arrowheads mark amoeboid and flagellate trophozoites, respectively. The assays were processed in three biological repeats (n = 3, mean ± SD). Significant differences were statistically measured by Student’s t-tests, with p< 0.05(*), p< 0.01(**), and ns, no significant difference. (TIF) [file ppat.1011891.s008.tif]

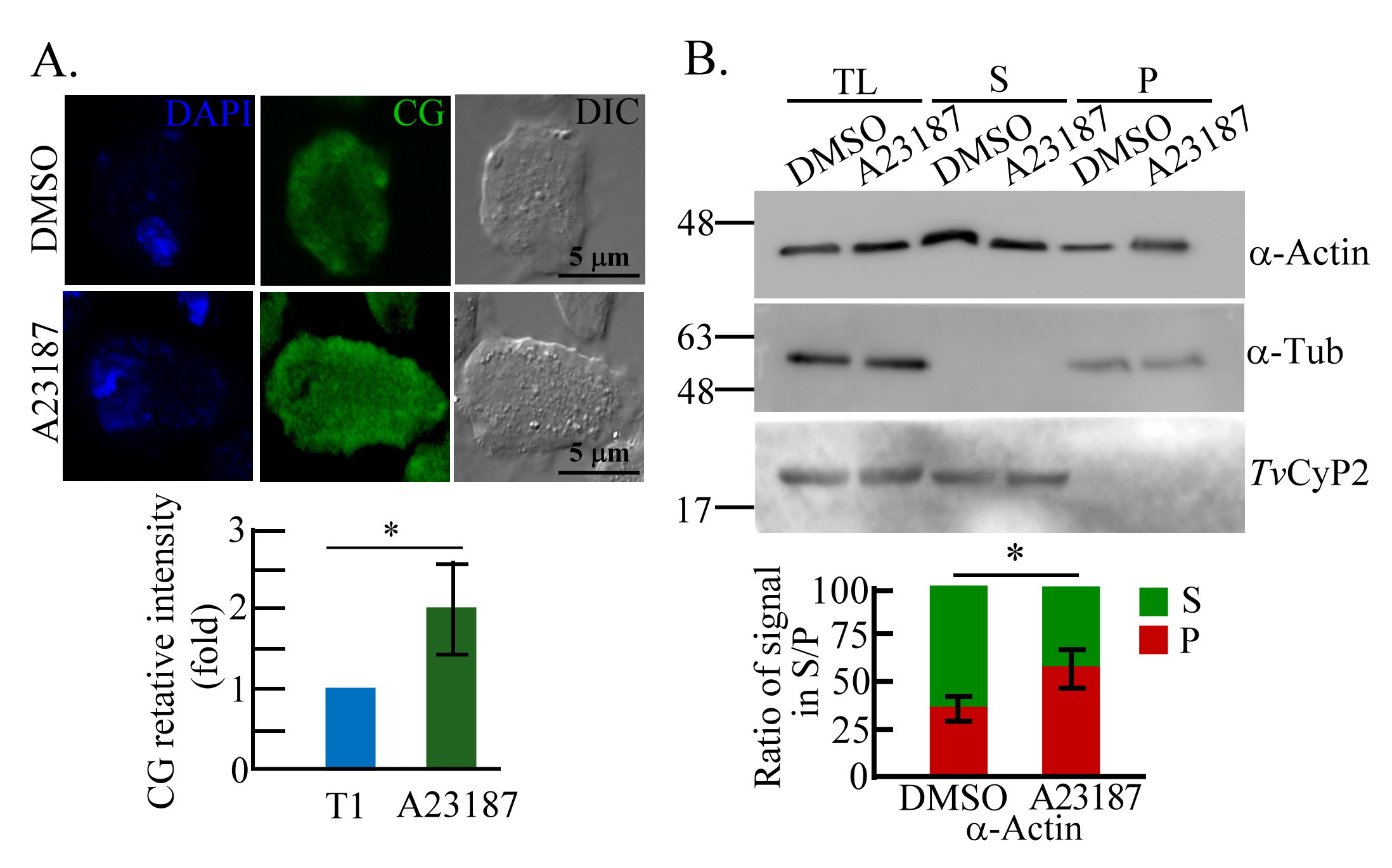

Supplement: S9 Fig — (A) The TH17 trophozoites treated with DMSO or A23187 were loaded with CG for confocal microscopy. The relative intensity of the CG signal was quantified in 300 trophozoites from five independent microscopic fields as shown in the bar graph (n = 3, mean ± SD). (B) The TH17 trophozoites pretreated with DMSO or A23187 were fractionated into supernatant and pellet fractions for western blotting. The ratio of α-actin signals in the supernatant (S) versus pellet (P) fractions was quantified, as shown in the bar graphs. The assays were processed in three biological repeats (n = 3, mean ± SD). Significant differences were statistically measured by Student’s t-tests with p< 0.05(*), p< 0.01(**), and ns, no significant difference. (TIF) [file ppat.1011891.s009.tif]

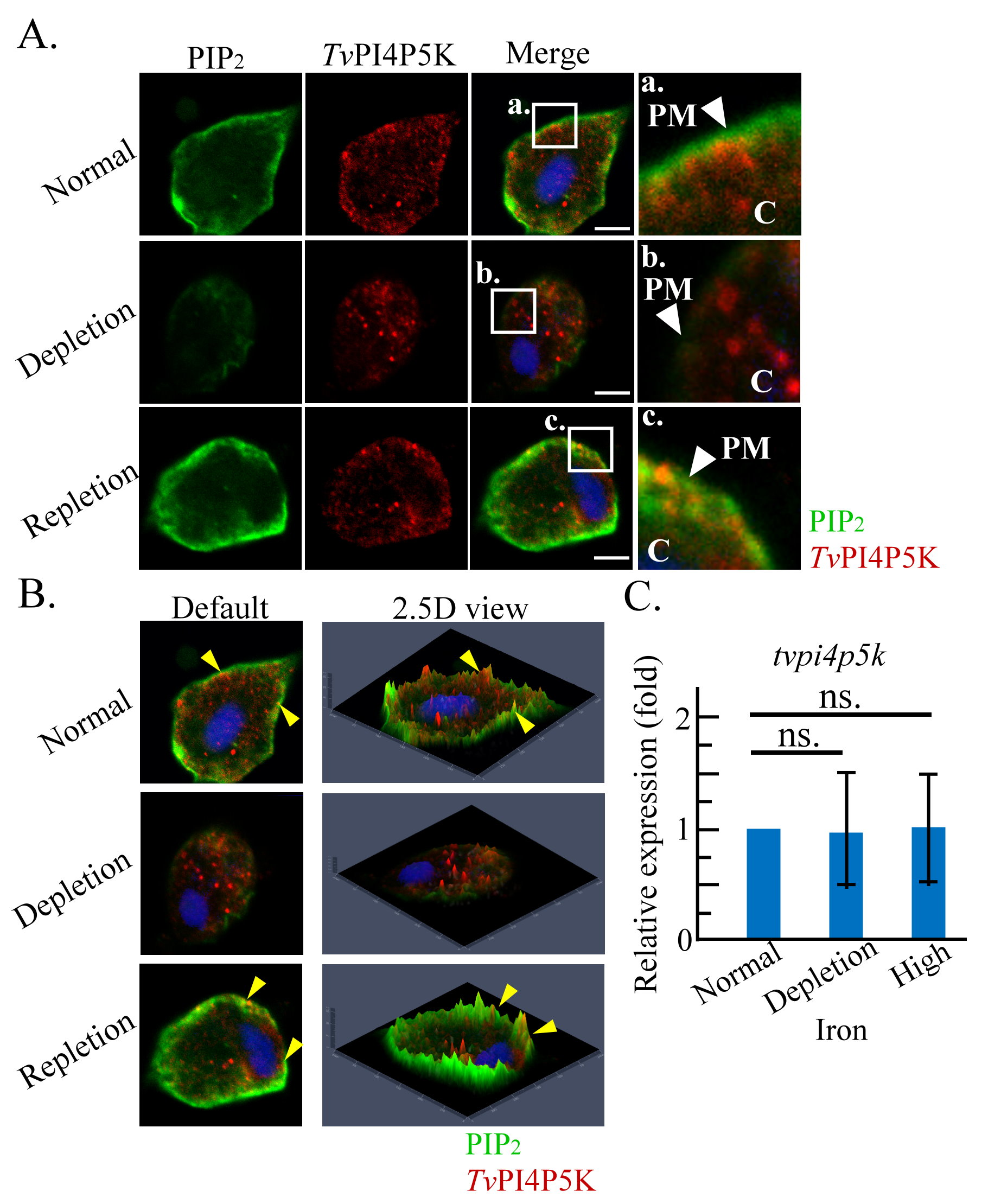

Supplement: S10 Fig — (A) The TH17 trophozoites cultured in normal-iron, iron-depleted, and iron-repletion medium overnight were fixed for IFA double staining for TvPI4P5K (red) and PIP2 (green) detection. The nuclei were stained with DAPI. The images boxed are magnified as shown in plots (a-c). Scale bar: 2 μm. White arrowheads indicate the parasite plasma membrane border (PM), and C indicates the cytoplasm. (B) The IFA images from (A) were viewed by 2.5D view of ZEN software. The yellow arrowheads indicate the partially colocalized signals (yellow) of PIP2 (green) and TvPI4P5K (red) in specific plasma membrane areas. (C) Transcription of the tvpi4p5k gene in T. vaginalis from various iron conditions was quantified by qPCR. The relative gene expression normalized to β-tubulin was shown in the bar graph (n = 3, mean ± SD). Significant differences were analyzed by Student’s t-tests, with p< 0.05(*) and p< 0.01 (**). (TIF) [file ppat.1011891.s010.tif]

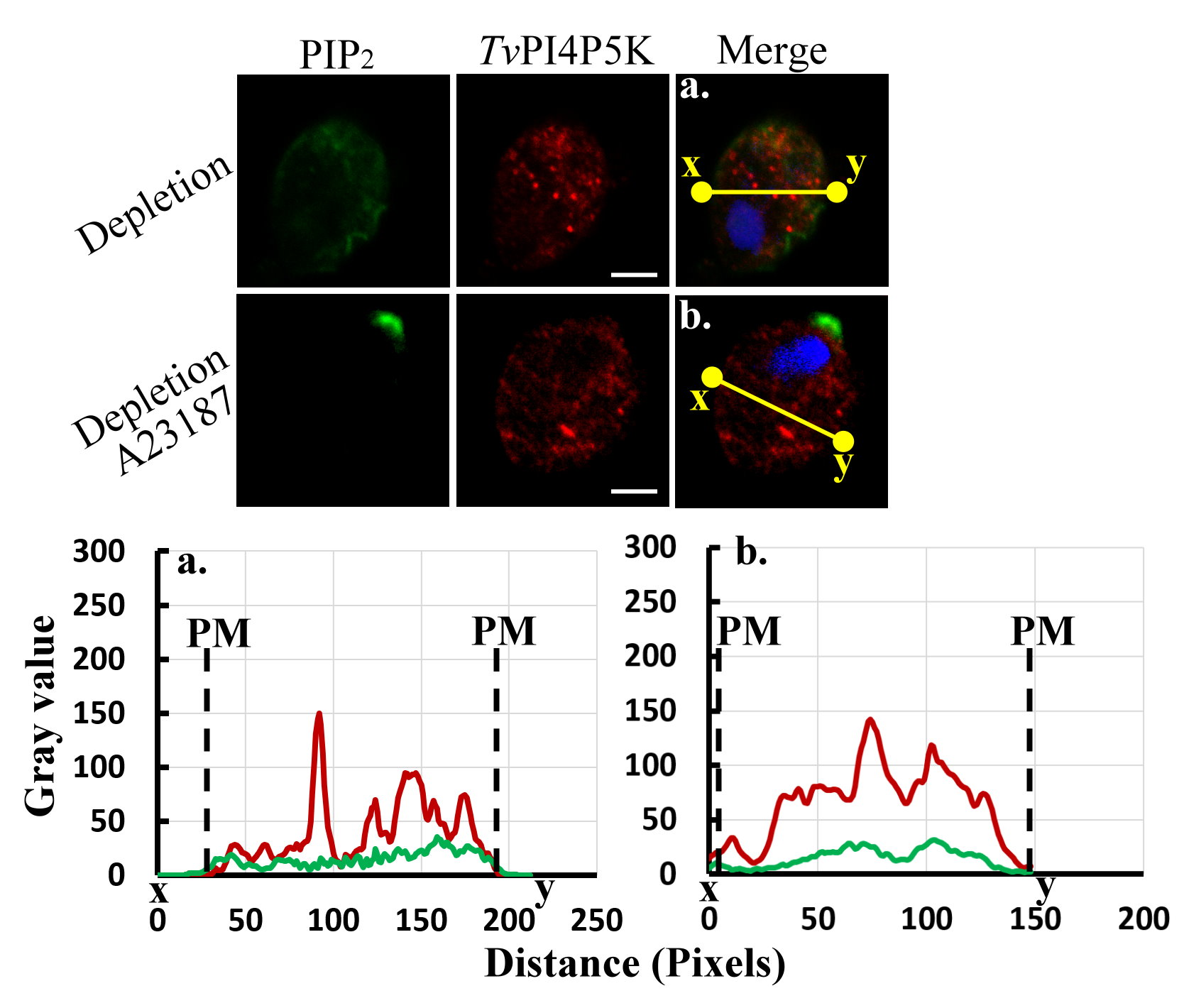

Supplement: S11 Fig — The iron-depleted TH17 trophozoites with or without the A23187 challenge were fixed for IFA double staining with anti-TvPI4P5K and anti-PIP2 antibodies. Nuclei were stained with DAPI. Scale bar: 2 μm. The signal intensity distribution on the yellow line between x and y sites in the representative micrograph (a, b) was analyzed by ImageJ as shown in the corresponding plots (a, b). PM indicates the plasma membrane boundary. (TIF) [file ppat.1011891.s011.tif]

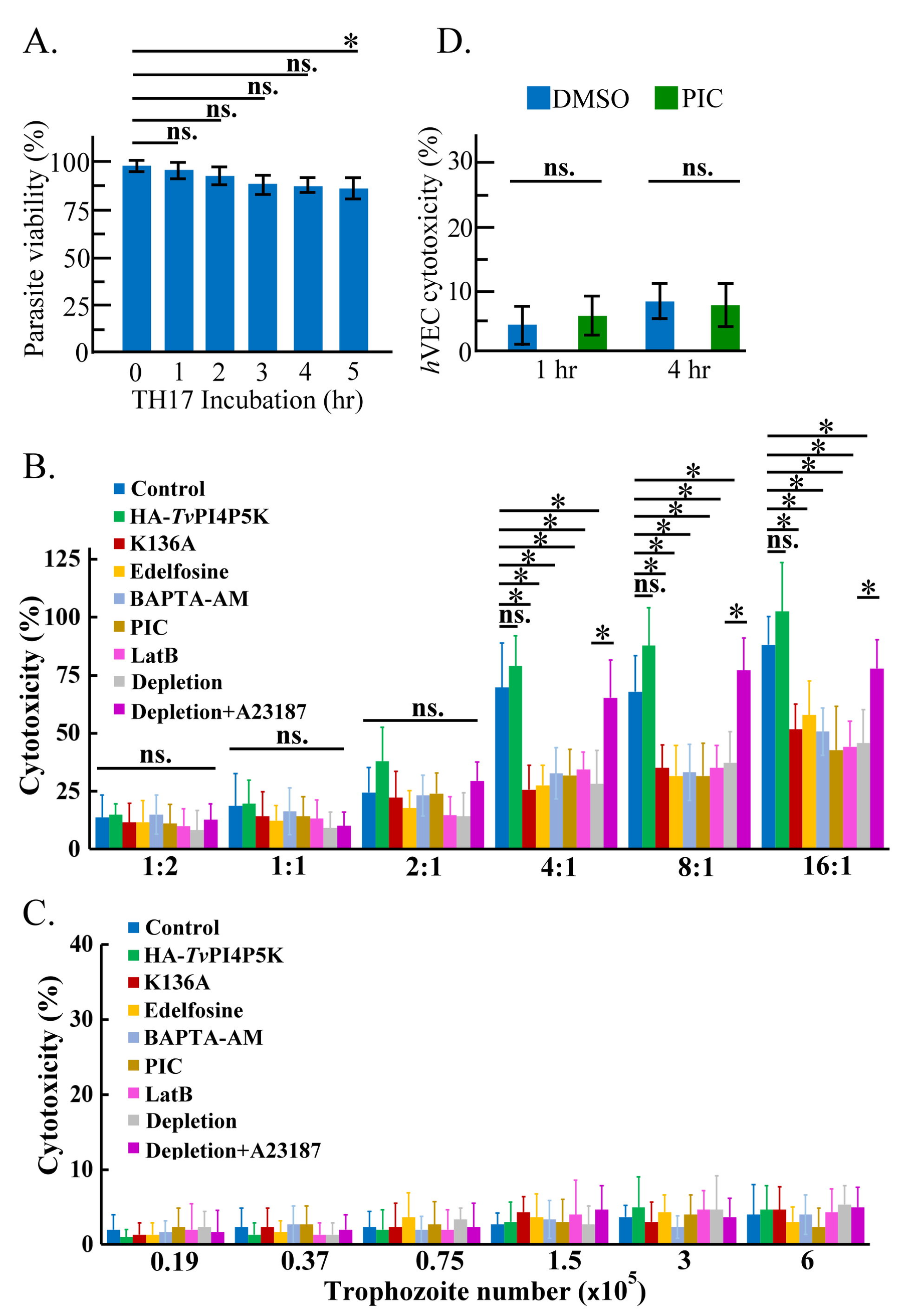

Supplement: S12 Fig — (A) The viability of TH17 trophozoites in a microplate with keratinocyte serum-free medium at 37°C in 5% CO2 was evaluated at different time points by the trypan blue exclusion assay. (B) The hVECs were co-cultured with the parasites with conditions as in Fig 8A at different MOI for 4 hr. The spent medium supernatant was collected for LDH cytotoxicity assay. (C) The parasites were pretreated as in Fig 8A. Different amounts of trophozoites (equivalent to the trophozoites number used in Fig 8A) were inoculated in keratinocyte serum-free medium for 1 hr at 37°C under 5% CO2. The spent medium supernatant was collected for LDH cytotoxicity assay. (D) The spent media from the parasite-free hVECs culture treated with DMSO or PIC for 1 or 4 hr were collected for LDH cytotoxicity assay. The assays were processed in three biological repeats (n = 3, mean ± SD), and significant differences were analyzed by Student’s t-tests, with p< 0.05(*) and p< 0.01 (**). (TIF) [file ppat.1011891.s012.tif]

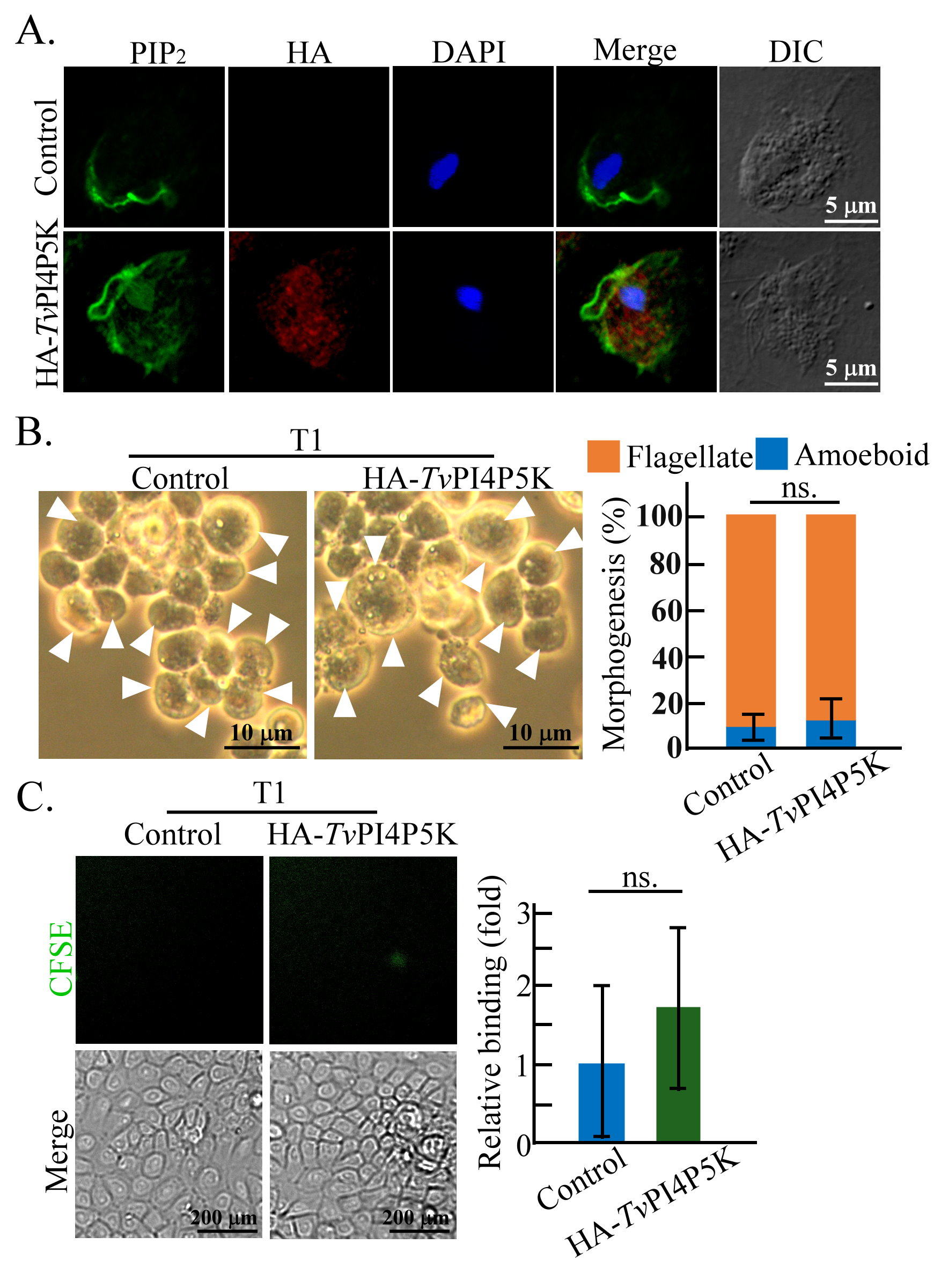

Supplement: S13 Fig — T1 trophozoites with or without HA-TvPI4P5K overexpression were sampled for IFA double staining with anti-HA or anti-PIP2 (A), morphogenesis assay (B), and cytoadherence assay (C). For (B), white arrowheads label flagellate trophozoites. The assay was processed in three biological repeats to measure the proportion of the flagellate versus amoeboid trophozoites, as shown in the bar graph (n = 3, mean ± SD). For (C), the CFSE-prelabeled parasites were co-cultured with hVECs for 1 hr. After washing, the bound parasites were detected by a confocal microscope. The assay was processed in three biological repeats, and the relative ratio of the bound parasite was quantified with the non-transgenic control defined as 1 (n = 3, mean ± SD). The significant differences were analyzed by Student’s t-tests, with p< 0.05(*), p< 0.01(**), ns. no significant difference. (TIF) [file ppat.1011891.s013.tif]
