# Supplementary material for: PIP2 regulating calcium signal modulates actin cytoskeleton-dependent cytoadherence and cytolytic capacity in the protozoan parasite Trichomonas vaginalis
Source: PLoS Pathog. 2023 Dec 18;19(12):e1011891. doi: 10.1371/journal.ppat.1011891 (PMC10758264; doi:10.1371/journal.ppat.1011891)
Supplement: S1 Data — This file includes raw image data of Figs 1C, 2A, 2C, 2G, 2H, 3F, 5A, 5B, 5C, 5D, 5E, 6A, 6B, 7D, S3C, S6 and S9B. (PDF) [file ppat.1011891.s016.pdf]

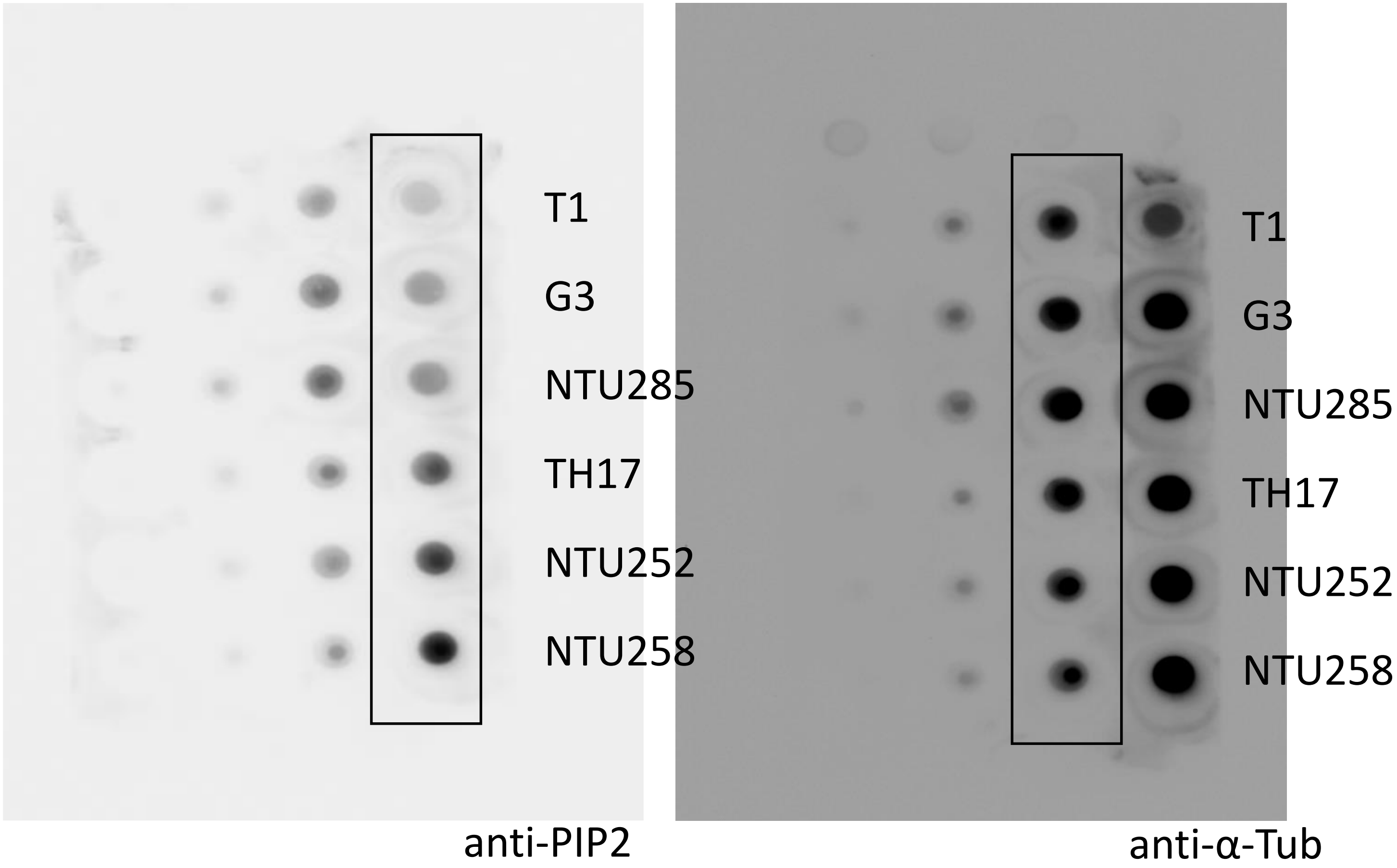

Fig 1C. The Raw data of dot blot assay. The region boxed was shown in this article.

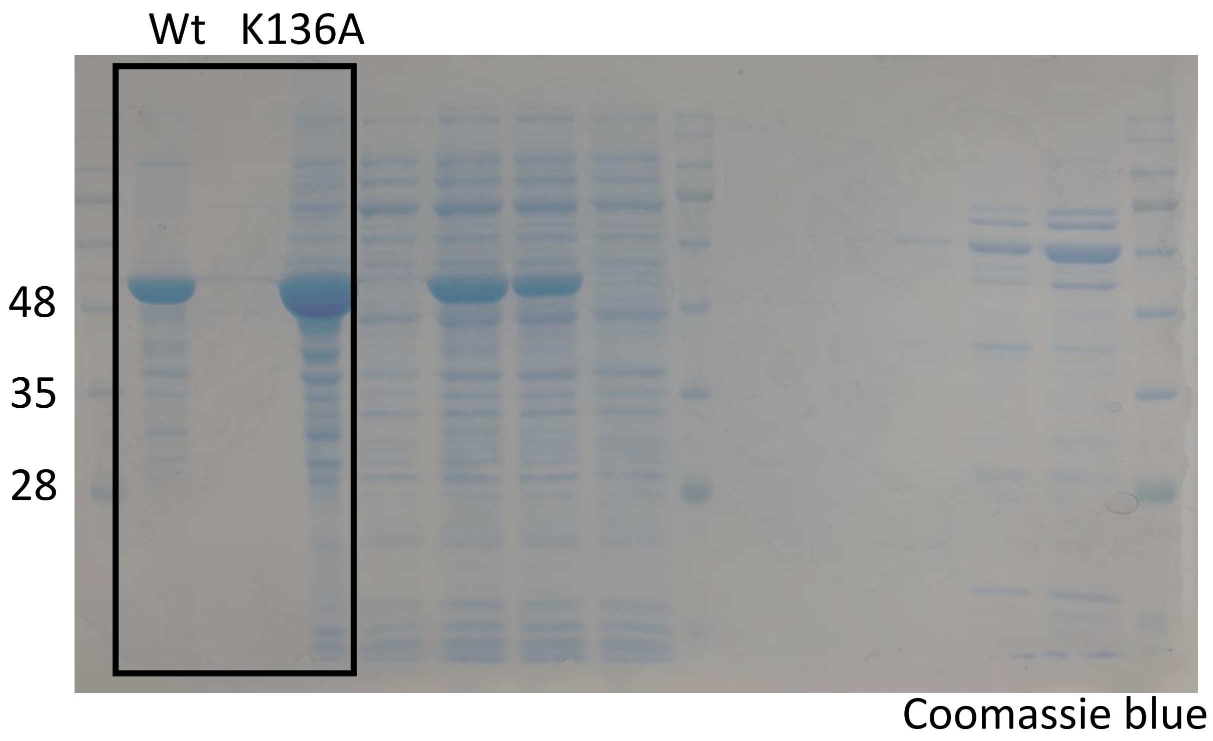

Fig 2A. The raw data of SDS PAGE with Coomassie Blue staining. The boxed regions were shown in the article.

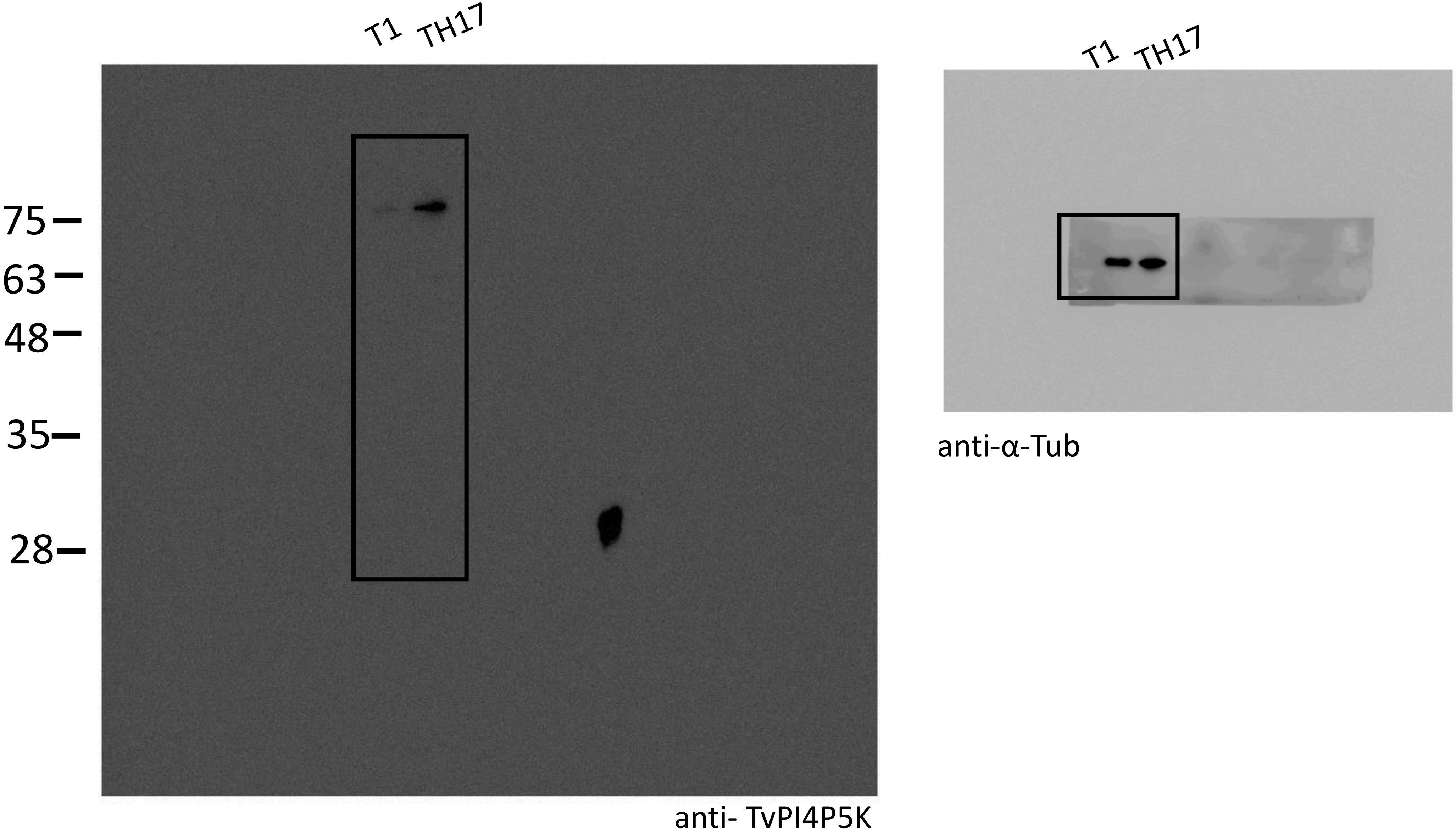

Fig 2C. The raw data of western blotting. The boxed regions were shown in the article.

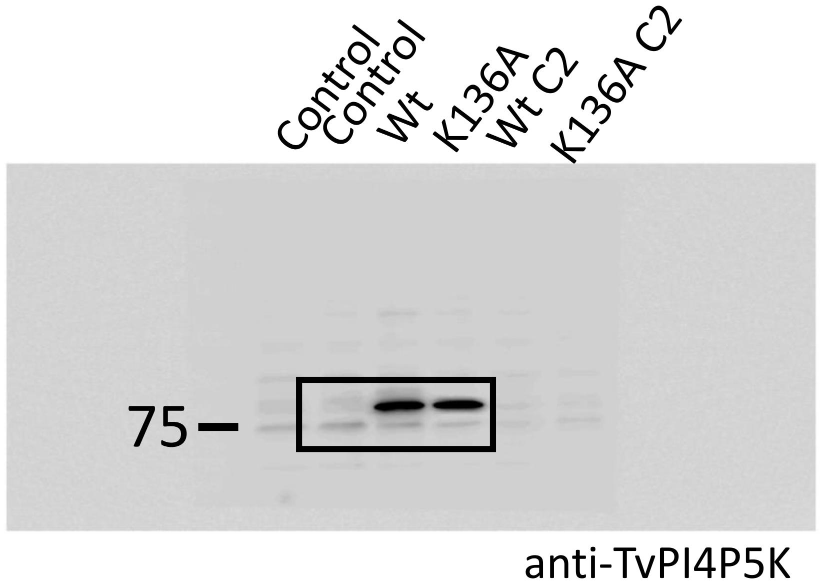

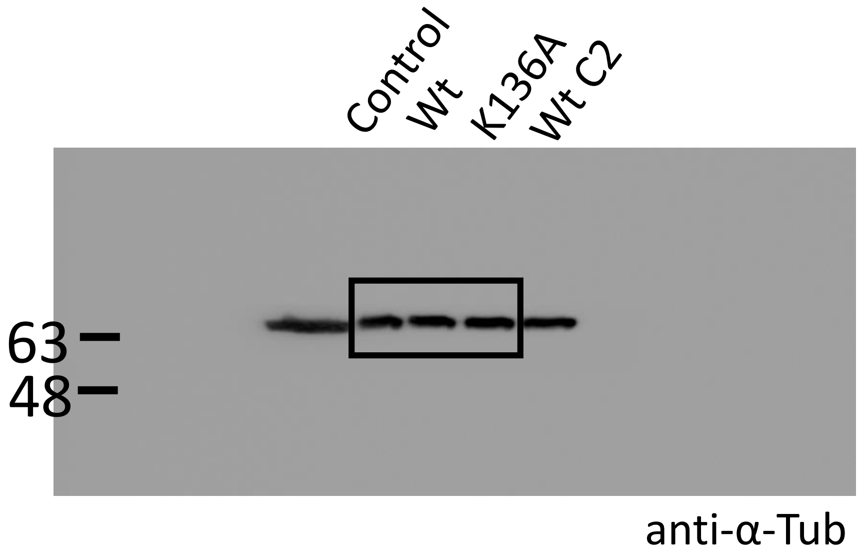

Fig 2G. The raw data of western blotting. The boxed regions were shown in the article.

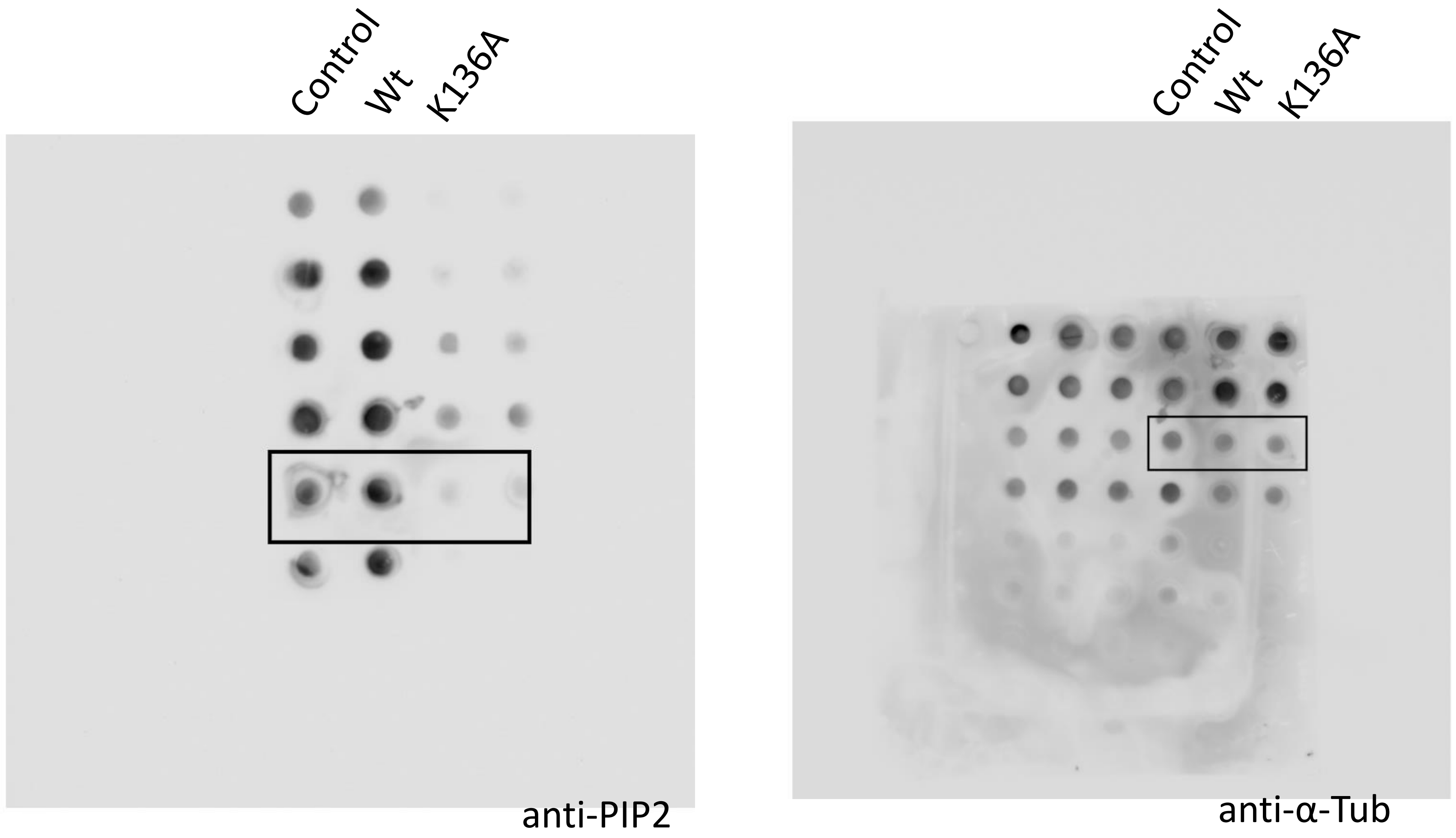

Fig 2H. The raw data of dot blot assay. The boxed regions were shown in the article.

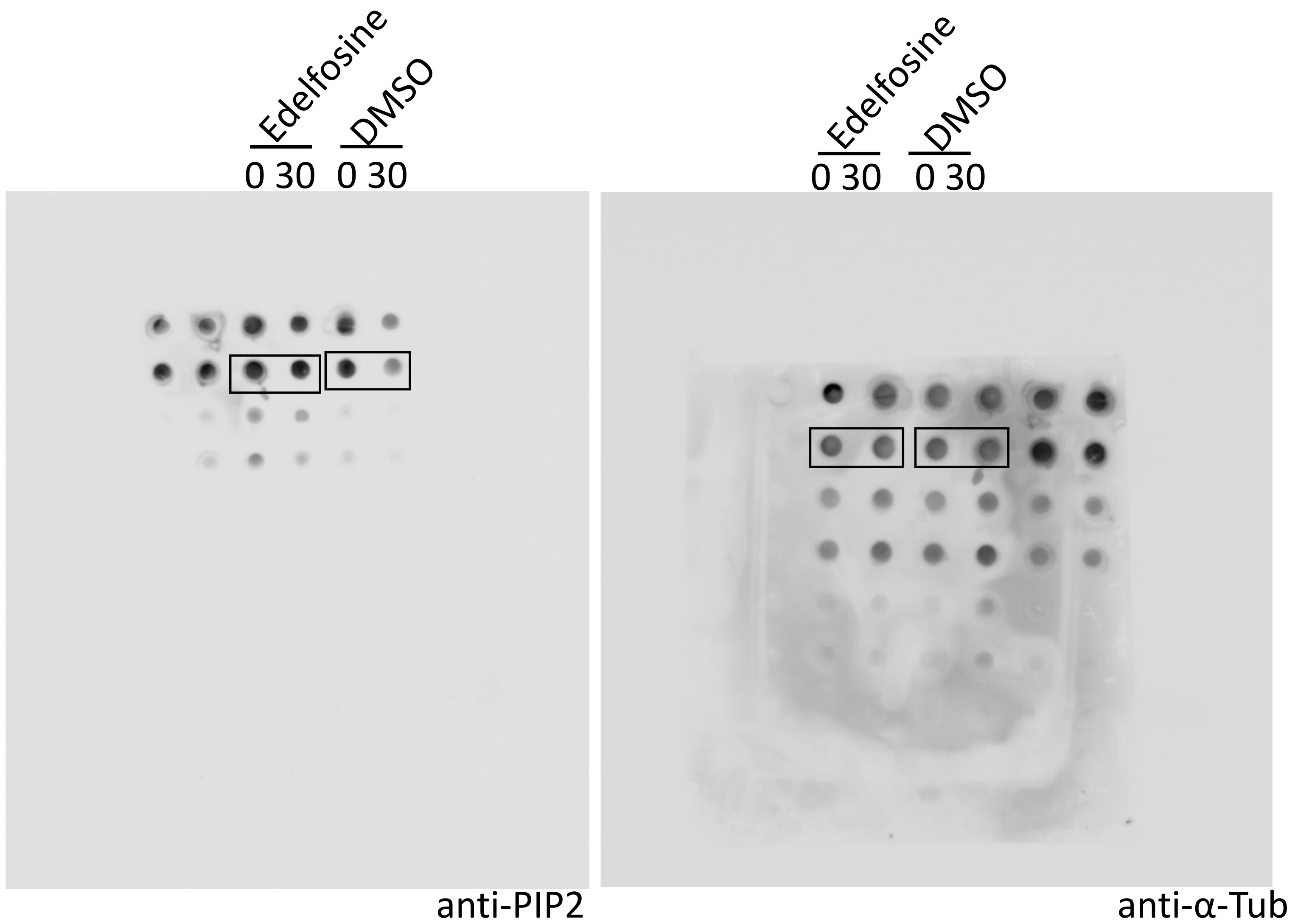

Fig 3F. The raw data of dot blot assay. The boxed regions were shown in the article.

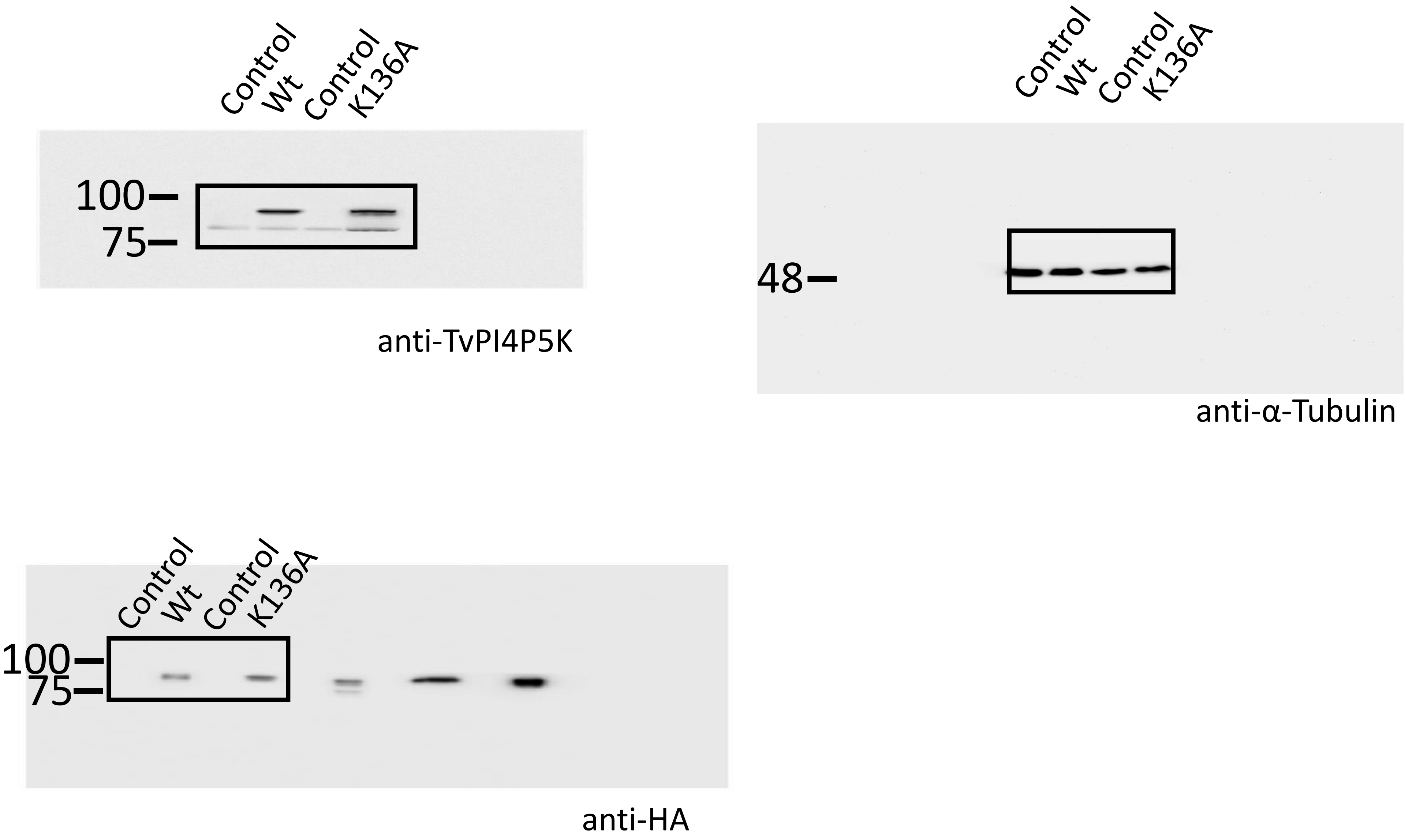

Fig 5A. The raw data of western blotting. The boxed regions were shown in the article.

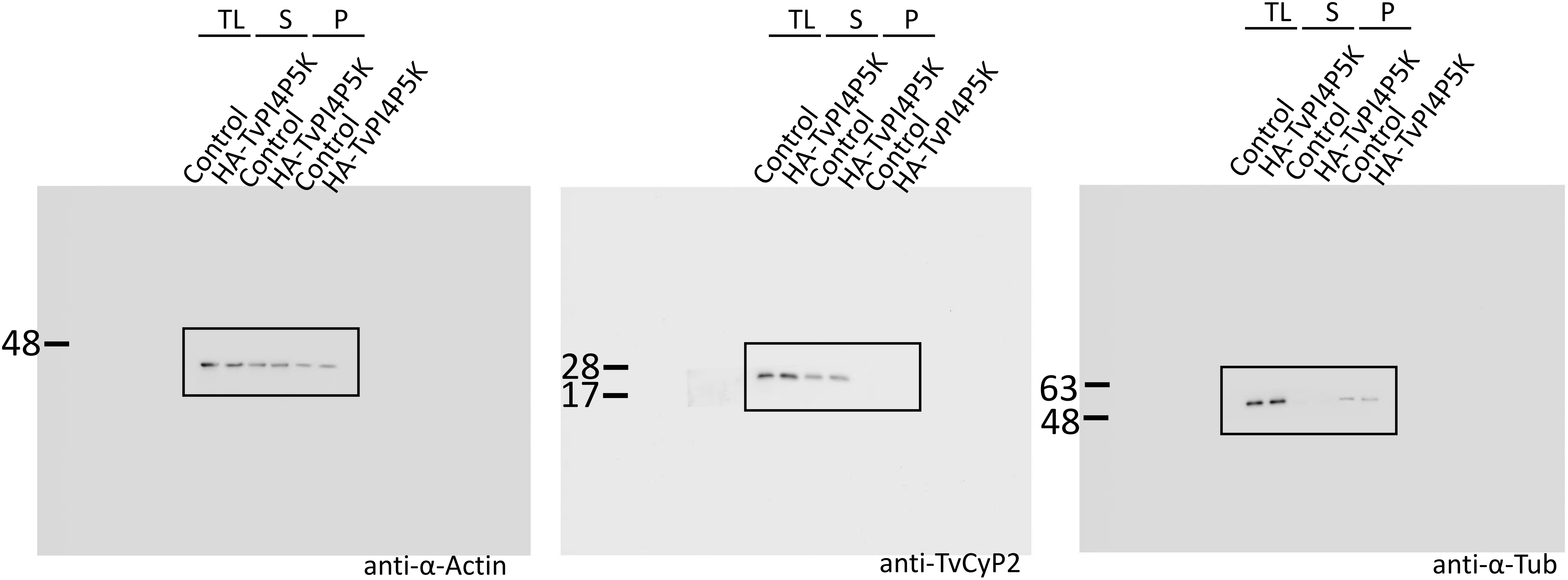

Fig 5B. The raw data of western blotting. The boxed regions were shown in the article.

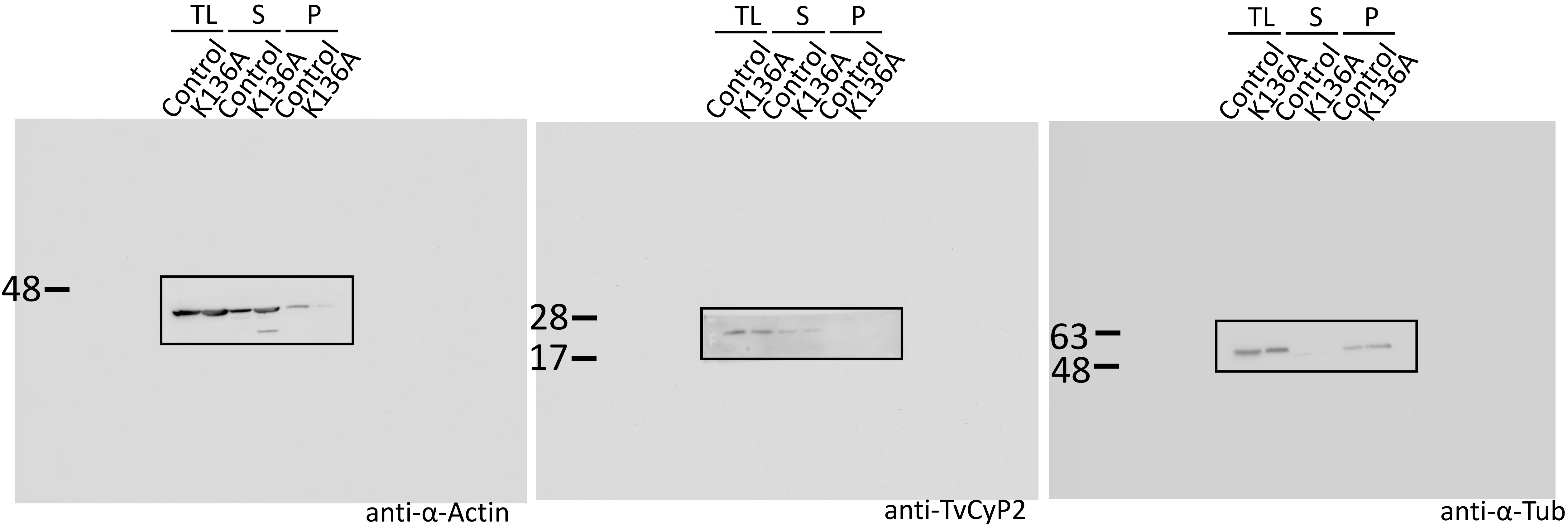

Fig 5C. The raw data of western blotting. The boxed regions were shown in the article.

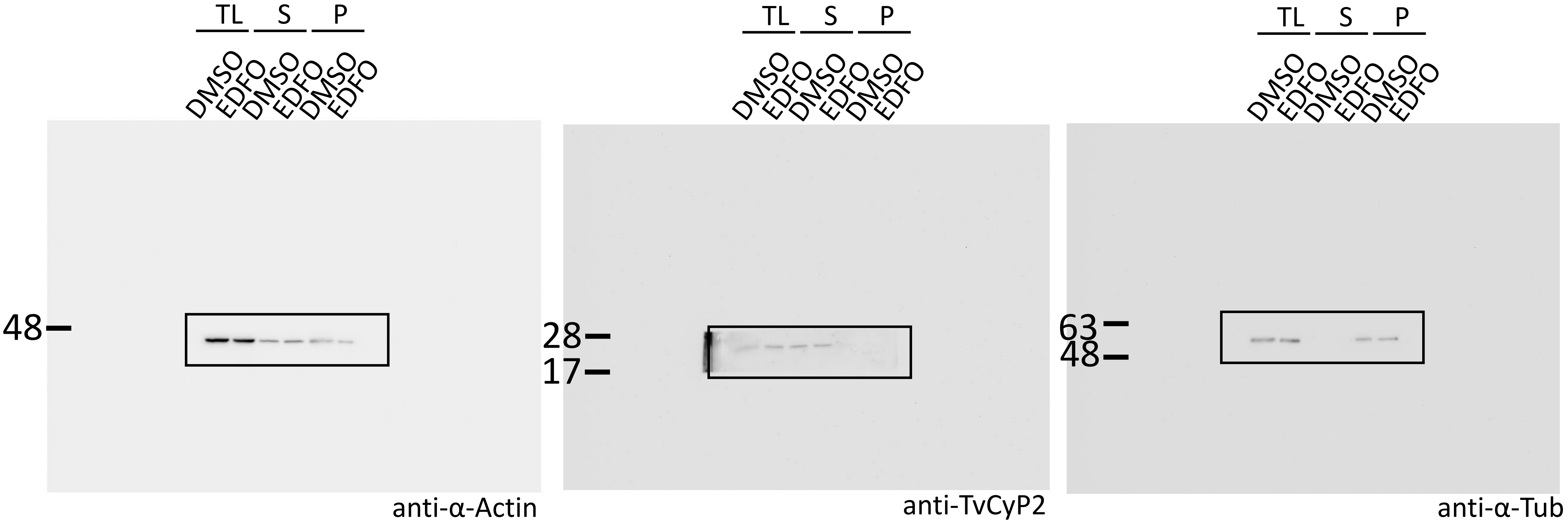

Fig 5D. The raw data of western blotting. The boxed regions were shown in the article.

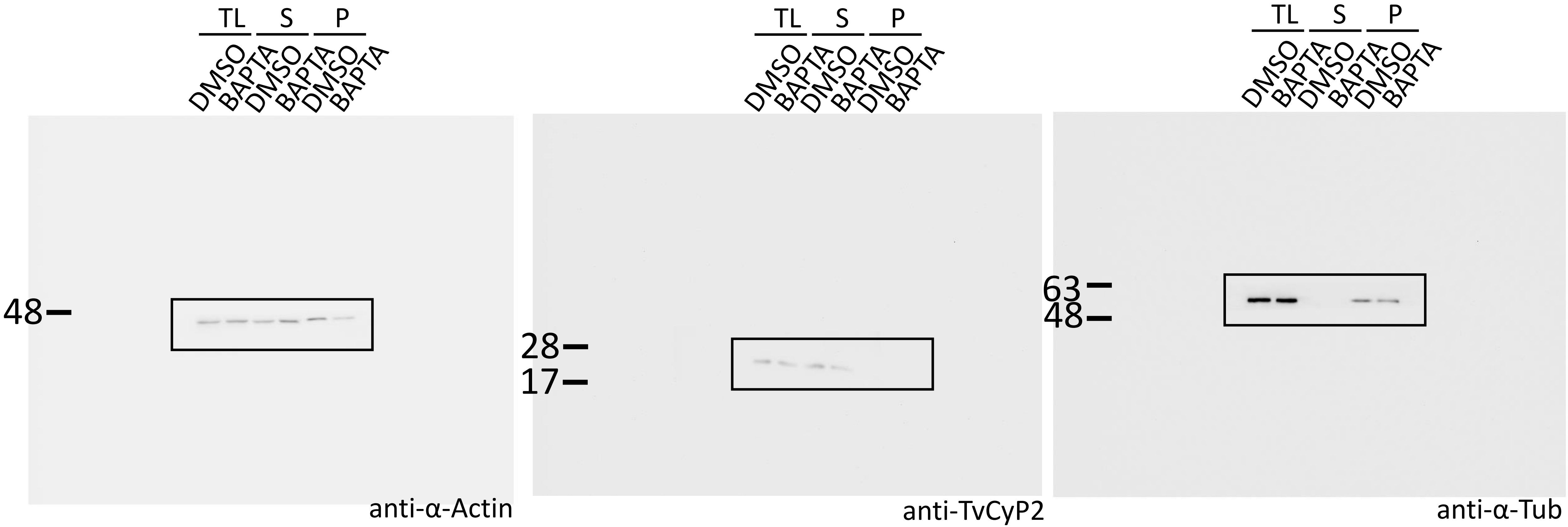

Fig 5E. The raw data of western blotting. The boxed regions were shown in the article.

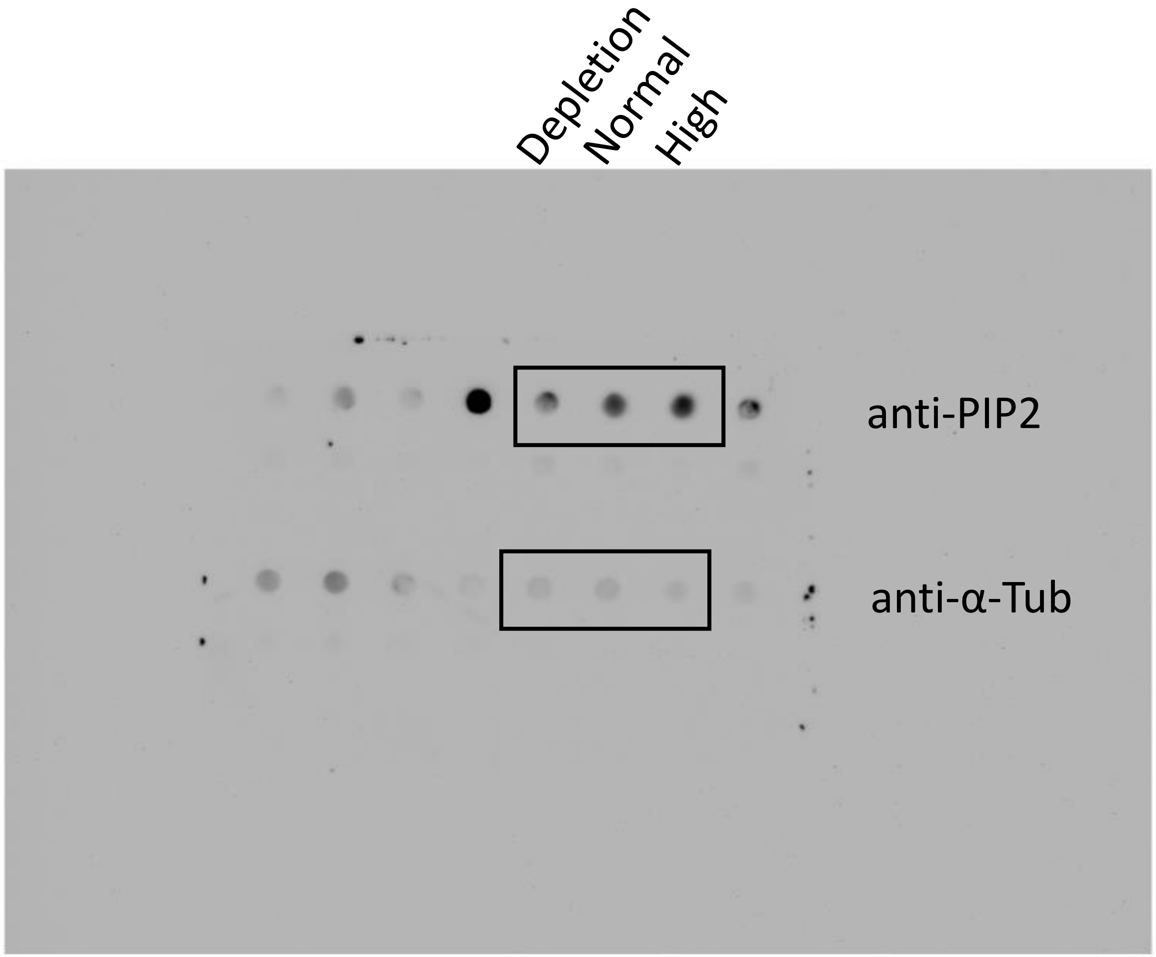

Fig 6A. The raw data of dot blot assay. The boxed regions were shown in the article.

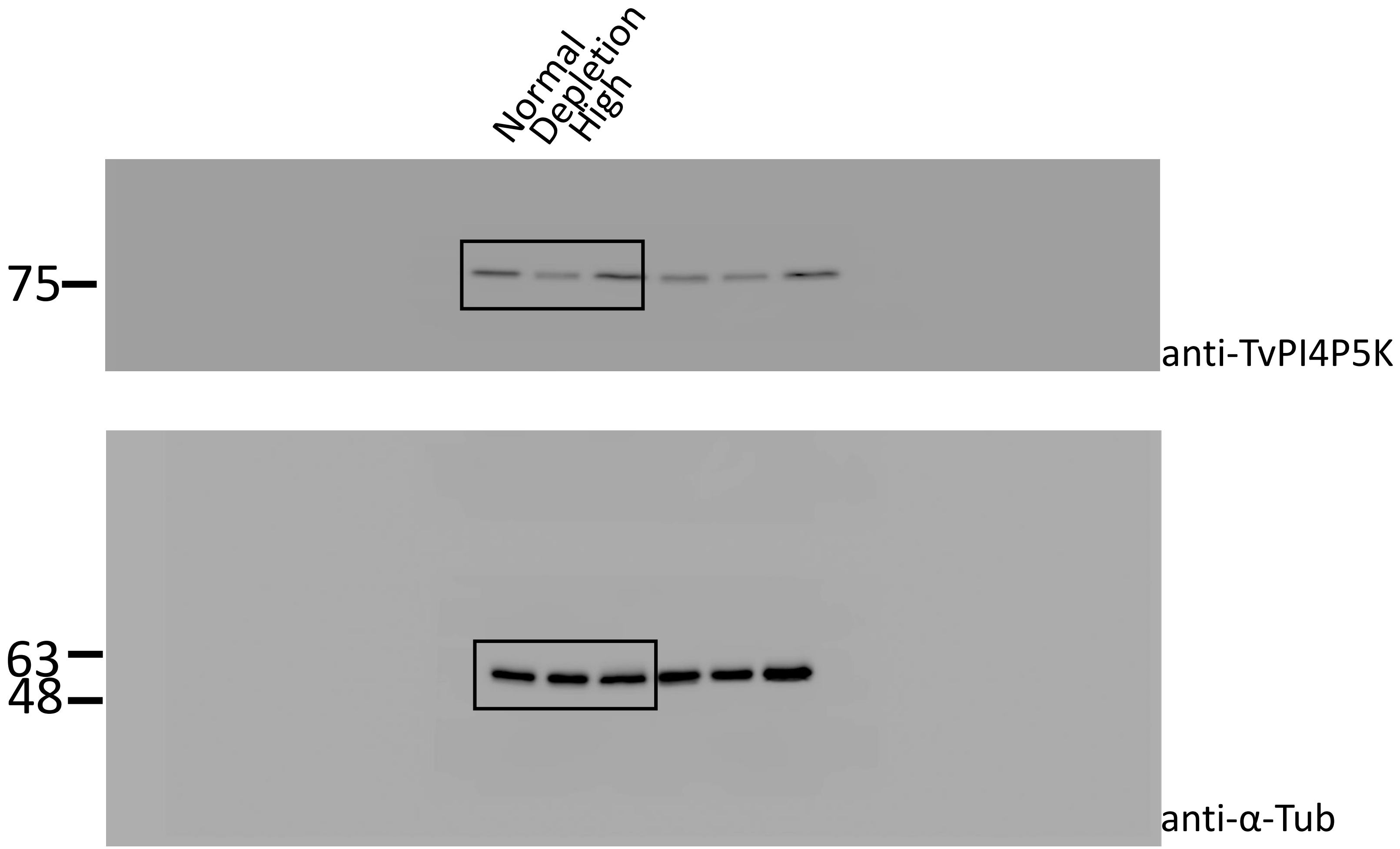

Fig 6B. The raw data of western blotting. The boxed regions were shown in the article.

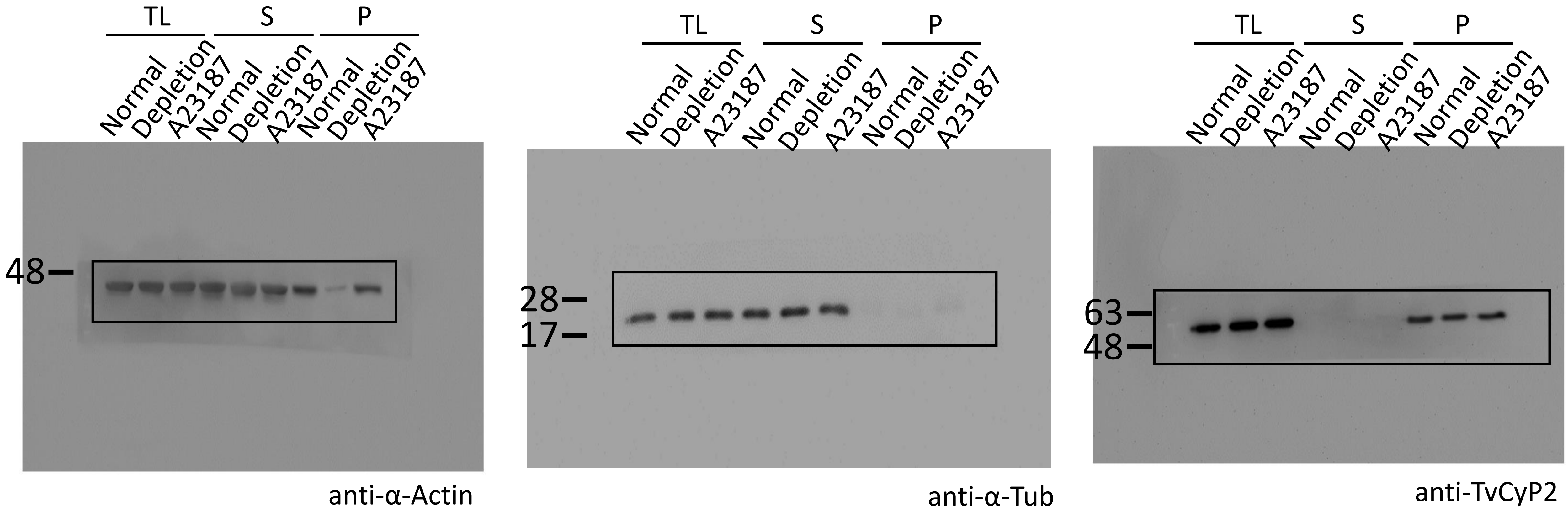

Fig 7D. The raw data of western blotting. The boxed regions were shown in the article.

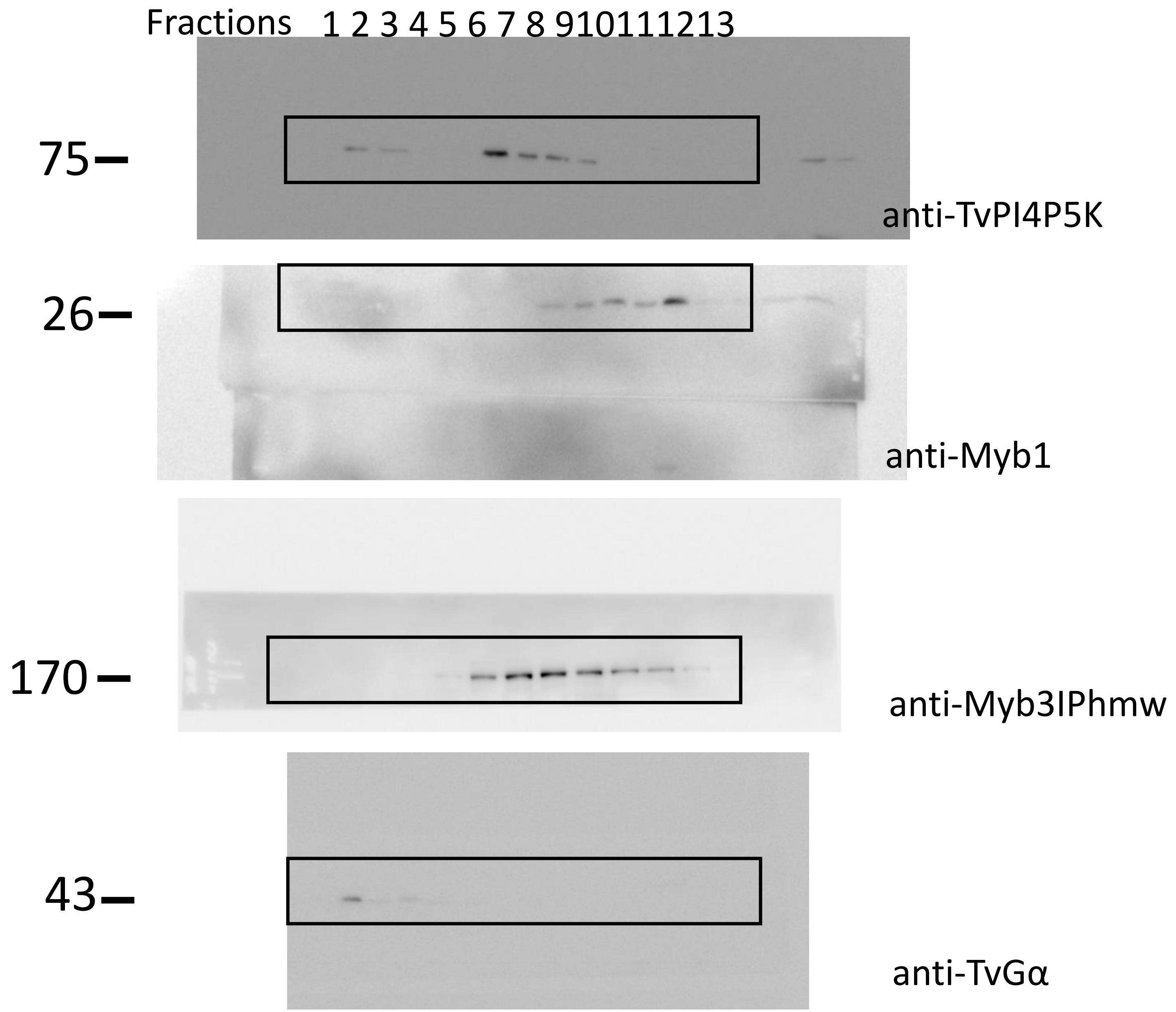

S3C Fig. The raw data of western blotting. The boxed regions were shown in the article.

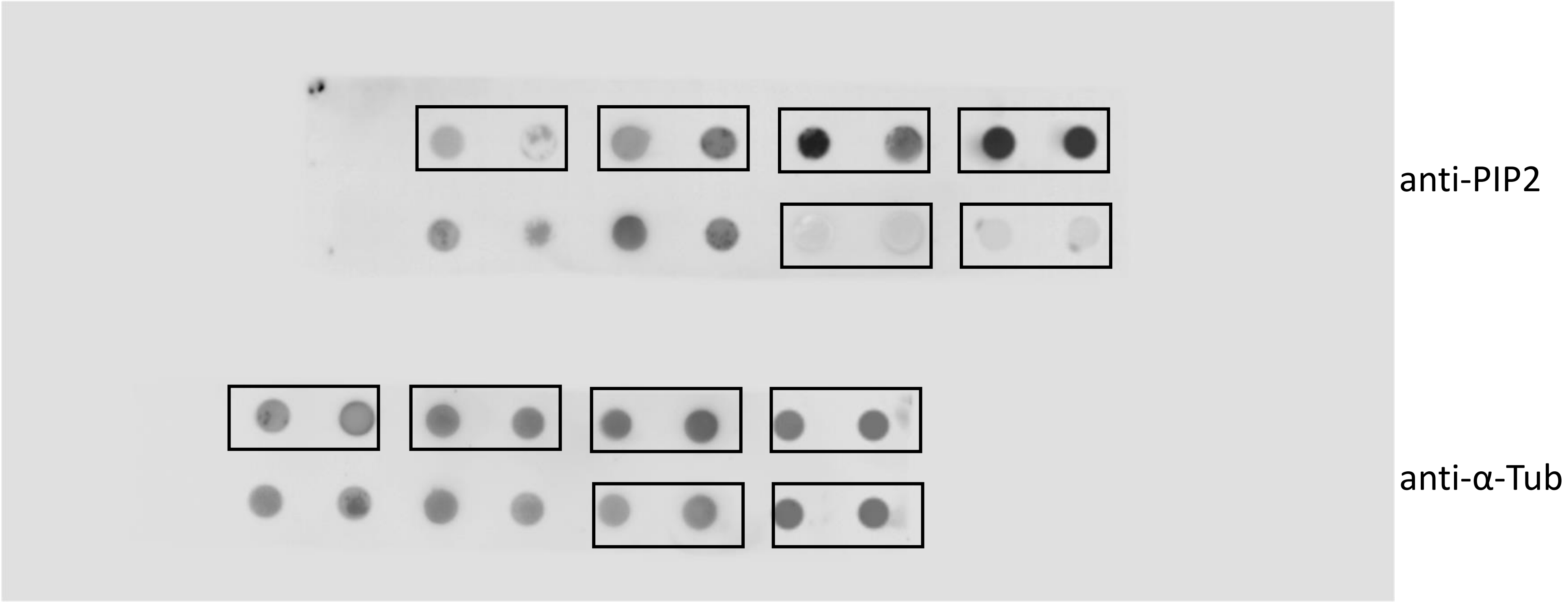

S6 Fig. The raw data of western blotting. The boxed regions were shown in the article.

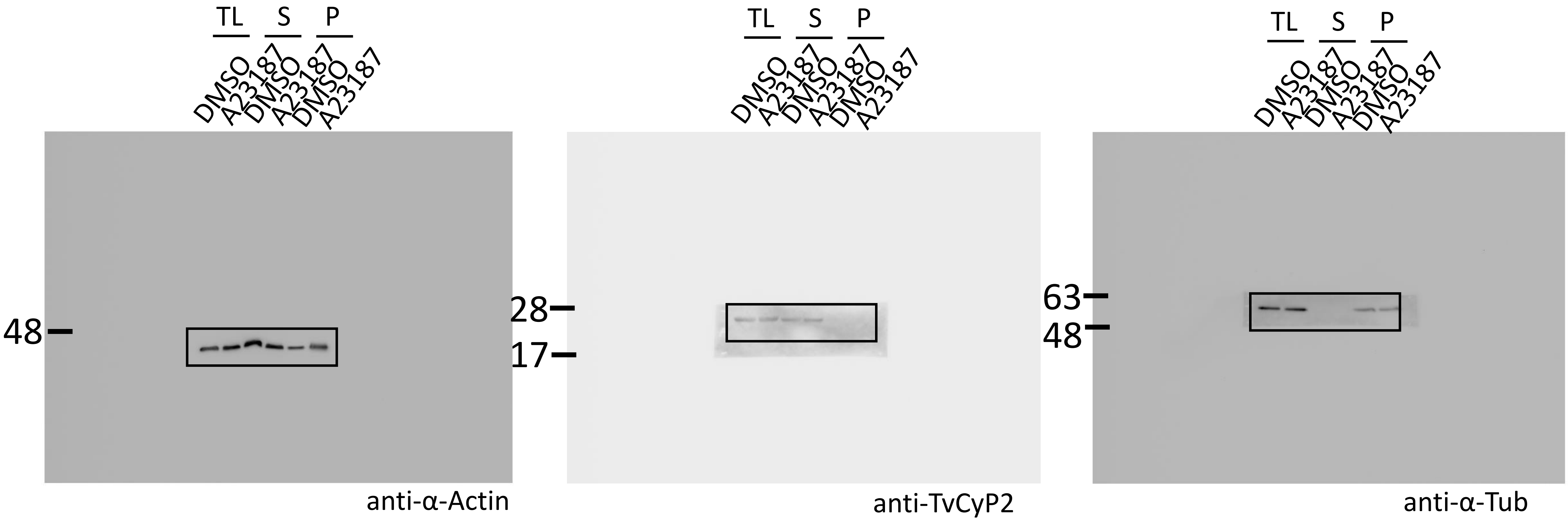

S9B Fig. The raw data of western blotting. The boxed regions were shown in the article.
