# Supplementary material for: PIP2 regulating calcium signal modulates actin cytoskeleton-dependent cytoadherence and cytolytic capacity in the protozoan parasite Trichomonas vaginalis
Source: PLoS Pathog. 2023 Dec 18;19(12):e1011891. doi: 10.1371/journal.ppat.1011891 (PMC10758264; doi:10.1371/journal.ppat.1011891)
Supplement: Attachment — Submitted filename: PIP2 PLOS Reviewers comments R1.pdf [file ppat.1011891.s018.pdf]

Dear Reviewers

We wish to express our gratitude for your valuable suggestions which made this manuscript more convincing and readable. In this revision, we have addressed most reviewers' concerns and supplied supportive information. Please see our responses to the comments below and the tracked modifications in the manuscript file.

Dear Dr Hsu,

Thank you for submitting your manuscript titled "PIP2 regulating calcium signal modulates actin cytoskeleton-dependent cytoadherence and cytolytic capacity in the protozoan parasite Trichomonas vaginalis" to PLoS Pathogens for consideration.

The manuscript was reviewed by members of the editorial board and three expert reviewers, whose full reviews are included at the end of this email.

In general, all reviewers welcome the data shown in this submission and consider it worthy of dissemination to the wider scientific community. However, two of the three reviewers list significant concerns with respect to some of the presented experimental approaches, the data derived from these and data interpretation.

Reviewer 1 questions the appropriateness of approaches to quantification of expression and makes concrete suggestions for improvement. Reviewer 3 is especially concerned with approaches to the monitoring of cytotoxicity and with experimental conditions for cytoadherence studies, a concern also shared by Reviewer 1.

Re: The approaches to quantification of expression were improved and experimental concerns of the cytotoxicity and cytoadherence assay were addressed in this revision as detailed below.

For these and other reasons related to clarity of text, usage of acronyms and figure labelling, the manuscript can be considered for publication only after major revision.

We cannot make any decision about publication until we have seen the revised manuscript and your response to the reviewers' comments. Your revised manuscript is also likely to be sent to reviewers for further evaluation.

When you are ready to resubmit, please upload the following:

[1] A letter containing a detailed list of your responses to the review comments and a description of the changes you have made in the manuscript. Please note while forming your response, if your article is accepted, you may have the opportunity to make the peer review history publicly available. The record will include editor decision letters

(with reviews) and your responses to reviewer comments. If eligible, we will contact you to opt in or out.
[2] Two versions of the revised manuscript: one with either highlights or tracked changes denoting where the text has been changed; the other a clean version (uploaded as the manuscript file).
Important additional instructions are given below your reviewer comments.
Please prepare and submit your revised manuscript within **60 days**. If you anticipate any delay, please let us know the expected resubmission date by replying to this email.
Please note that revised manuscripts received after the 60-day due date may require evaluation and peer review similar to newly submitted manuscripts.
Thank you again for your submission. We hope that our editorial process has been constructive so far, and we welcome your feedback at any time. Please don't hesitate to contact us if you have any questions or comments.
Sincerely,

Carmen Faso, Ph.D.
Academic Editor
PLOS Pathogens

James Collins III
Section Editor
PLOS Pathogens

Kasturi Haldar
Editor-in-Chief
PLOS Pathogens
orcid.org/0000-0001-5065-158X

Michael Malim
Editor-in-Chief
PLOS Pathogens
orcid.org/0000-0002-7699-2064

****

Reviewer's Responses to Questions

Part I - Summary

Please use this section to discuss strengths/weaknesses of study, novelty/significance,

general execution and scholarship.

Reviewer #1: TV is the most common cellular pathogen sexually transmitted worldwide and it is particularly common among resource limited populations. TV infections are strongly associated with higher transmission rate of HIV and a number of other important health sequelae including low birth weight, premature rupture of membranes and preterm. However, we still know very little about the pathogenesis of this common parasite. Here, the authors demonstrate that PIP2 hydrolysis followed by intracellular calcium rising is vital for parasite amoeboid morphogenesis and cytoadherence. I consider that there is valuable information on the paper that will likely be of interest to the T. vaginalis community because there is still very little known about the molecular basis of host interaction for this parasite. However, the some of the data presented and its interpretation require modifications prior to publication. The resubmission of the manuscript is encouraged after major modification.

Reviewer #2: This work satisfies the PLOS Pathogens criteria for publication: The manuscript by Chen et al., entitled “PIP2 regulating calcium signal modulates actin cytoskeleton-dependent cytoadherence and cytolytic capacity in the protozoan parasite Trichomonas vaginalis” is an interesting piece of data that includes correctly design experiments, clean result presentation, and great novelty for the field of host-parasite interaction. In this work, the authors investigated in deep the link between the cytoskeleton and PIP2 signaling in T. vaginalis and the possible relationship with environmental iron response. This study identified differential PIP2 and TvPI4P5K expression in adherent and nonadherent Tv isolates and analyzed their functional roles in the cytopathogenicity of Tv. Their data reveal the biological significance of PIP2 signal transduction in regulating T. vaginalis pathogenicity.

Reviewer #3: The authors investigated PIP2 distribution in a human parasite Trichomonas vaginalis, its role as a second messenger, the role of phosphatidylinositol-4-phosphate 5-kinase (TvPI4P5K), calcium and iron in flagellate-amoeboid morphogenesis, and relationship to the cell virulence (adherence, cytotoxicity). They showed that PIP2 localizes in the undulating membrane of non-adhering cells (T1), while on the plasma membrane of adhering cells (TH17). They confirmed the expected activity of TvPI4P5K and claimed that it colocalized on the plasma membrane with PIP2 in adherent cells in which higher expression was observed, while TvPI4P5K was in the cytosol of nonadhering cells (T1). They further reported that expression of mutated HA-tagged TvPI4P5K has a dominant negative effect on trichomonad's ability to transform to amoeboid form, adherence to hVEC cells, intracellular calcium level, and polymerization of actin. Moreover, iron depletion conditions were shown to affect the localization of PIP2, TvPI4P5K, and calcium. Finally, the authors claimed that

expression of HA-tagged TvPI4P5K stimulated cytotoxicity, while it was reduced in K136A mutant. The study is of moderate technical quality, the experiments are well-controlled and easy to follow, however, some conclusions are not supported by the data.

Part II – Major Issues: Key Experiments Required for Acceptance
Please use this section to detail the key new experiments or modifications of existing experiments that should be absolutely required to validate study conclusions.

Generally, there should be no more than 3 such required experiments or major modifications for a "Major Revision" recommendation. If more than 3 experiments are necessary to validate the study conclusions, then you are encouraged to recommend "Reject".

Reviewer #1: Specific comments
1. In order to make it easier to follow the rational behind the experiments, it would be nice to include schematic illustration of the complete PIP2 pathway. Without this illustration the results are difficult to follow.
Re: A scheme illustrating the PIP_2 pathway identified in this study was inserted in the revised Fig. 9.

2. Fig 1E: Quantification needs to be done using a more quantitative method such as quantitative PCR (qPCR) or similar.
Re: Quantitative PCR (qPCR) was exploited to quantify the relative transcription of the *tvpi4p5k* gene between isolates (Fig. 1D, line 133). The protocol was described in line 624.

3. Page 13, line 171: ¨HA-TvPI4P5K Wt and K136A were five-fold overexpressed in TH17 trophozoites (Fig. 2G)¨. Fig 2G is an immunoprecipitation experiment. It is not the appropriate experiment to measure expression. As example, it could be the case that endogenous protein could be forming complex but not the HA-tag version, then, you can pull down more HA-TvPI4P5K due to an accessibility issue.
Re: The western blotting procedure was modified to detect endogenous *Tv*PI4P5K expression in the *T. vaginalis* total cell lysates. Double amounts of protein lysate and a high-sensitivity Femto Chemiluminescent substrate (Visual Protein) were incorporated into the western blotting protocol (line 683). The endogenous and recombinant *Tv*PI4P5K expressions detected in the total lysate are shown in Fig. 2G.

4. Fig 2G: the image from the anti-Tv PI4P5K antibody was cut and the signal cannot be visualized properly. Please, modify and show the complete image to allow a better interpretation.

Re: The original data was replaced with endogenous *Tv*PI4P5K and recombinant HA-*Tv*PI4P5K signals in the *T. vaginalis* total lysates (Fig. 2G).

5. Page 16, line 217: ¨~80% trophozoites of TH17 non-transfectant or HA-TvPI4P5K transfectant transformed into amoeboid form but only ~40% in K136A mutant¨. Similar results were obtained when adherence was evaluated. I understand that no difference in amoebic transformation and adherence is observed when HA-TvPI4P5K is overexpressed probably because TH17 is already an adherent strain. To validate the obtained results, it would be nice to evaluate if there is a difference in amoebic transformation and parasite adherence when HA-TvPI4P5K is overexpressed in G3 or NTU285 less adherent isolates

Re: When HA-*Tv*PI4P5K overexpression increased the PIP_2 level in the less adherent T1 isolates, it only slightly changed the activities of amoeboid transformation and cytoadherence (S13 Fig.), suggesting that the PIP_2 might be a factor essential for the adherent isolate but not sufficient to regain actin-based activities in a less adherent isolate. However, the details remain to be investigated. The data was described in line 498.

6. In order to confirm that amoebic transformation induce PIP2 hydrolysis, it would be important to evaluate the level of PIP2 (similar as Fig 3F) using parasites transfected with HA-TvPI4P5K and K136A compared to control (experiment Fig 3B)

Re: The PIP_2 changes in the HA-*Tv*PI4P5K and K136A transfectants with or without Edelfosine treatment undergoing amoeboid transformation were evaluated by dot blot (S6 Fig.). The data are described in line 221.

7. Fig 5A: Why endogenous TvPI4P5K is not detected in the IP when using transfected parasites? (Although the figure was cut, it seems that endogenous protein was detected in Fig 2G)

Re: To directly detect the endogenous *Tv*PI4P5K expression in the parasite total lysates, double amounts of protein lysate and a high-sensitivity Femto Chemiluminescent substrate (Visual Protein) were incorporated into the western blotting protocol (line 683). The endogenous or recombinant *Tv*PI4P5K expression was detected in the parasite total lysates (Fig. 5A) and described in line 306.

8. Page 26, line 333: ¨The dot blot (Fig. 6A) and western blotting (Fig. 6B) revealed that the PIP2 and TvPI4P5K levels similar in the normal-iron or iron-replete parasites were higher than the parasites depleted of iron, showing that iron content in the normal growth medium sufficiently affects TvPI4P5K and PIP2 expression in T. vaginalis¨. This is a similar comment as before. I think that an IP is not the correct experiment to evaluate expression. Although a lower expression might be a possibility, there might be also alternative explanations: iron might be affecting the complex formation that might be altering the accessibility of the protein to the antibody affecting the results of the IP. The expression of TvPI4P5K should be demonstrated in the total extract, the IP might be affected by other circumstances. Based on these results, I don´t agree with this statement.

Re: As per the reviewer's comments, the western blotting was improved to detect endogenous *Tv*PI4P5K expression in the total parasite lysates (Fig. 6B). The modified western blot procedure was detailed in the materials and methods of the revised manuscript (line 683).

9. Page 26, line 342: “suggesting that iron very likely modulates TvPI4P5K expression…”. To demonstrate that TvPI4P5K gene expression is affected in iron-depleted, and iron-replete growth media, a qPCR analysis must be included. It is an easy experiment that can help to demonstrate if expression is affected by iron.

Re: The iron-induced endogenous *Tv*PI4P5K expression was detected by western blotting in the parasite total lysate. Meanwhile, qPCR revealed the *tvpi4p5k* gene transcription was barely regulated by environmental iron, suggesting that iron may regulate *Tv*PI4P5K expression at the level of translation or post-translation (S10 Fig.C), and the result was described in line 344. The mechanism is under investigation.

10. Fig 8: What does “The conditional parasites” means? was this experiment performed with parasites or conditioned media? I am not sure if I am understanding the experiment correctly, I assume that the experiment was performed using parasites.

Re: In Fig. 8, "The conditional parasites" means the parasites from different pretreatment conditions. To avoid confusion, the word "conditional" was removed from the sentence, and the pretreatment conditions were stated in the figure legend.

11. I am surprised to see that cytolysis experiments were performed in 1 h incubation. Usually, to evaluate cytolysis, the parasites are incubated with host cells for longer times (minimum 4 h in Lustig et al 2013, 8 h in Bastida Corcuera et al. 2005). The level of cytolysis (even when using highly cytolytic strains) is usually very low after 1 h incubation. What is the level of cytolysis observed in these experiments? Fig 8A is

normalized and does not allow to evaluate the level of cytolysis. It might be worthy to perform this experiment using longer incubation time to increase cytolysis. Also, as control, it is important to evaluate the cytolysis of host cells when incubated with the correspondent media plus the different drugs (without parasites). It is important to include this control as these drugs might have a direct effect in the host cells

Re: TH17 is a laboratory strain, and the trophozoites were expanded, aliquoted, and cryopreserved since the initial isolation. When only the freshly reactivated (no longer than one month) TH17 trophozoites were used in each assay, they may retain more intact cytotoxic activity. Based on our preliminary conditional tests, the TH17 isolate might be more oxygen-sensitive and start to die (aggregated and suspended) post-culture in a microplate with keratinocyte serum-free medium in the 5% CO_2 over 5 hr (S12 Fig. A, line 402). Therefore, we focused on the early 1-hr cytotoxic effects to rule out uncertain influences of the dead parasites. A 4-hr cytotoxicity assay was supplied in S12 Fig. B and described in line 409 as per the reviewer's comment.

For Fig. 8A, the cytotoxicity was evaluated by calculating the OD_{490} ratio of *h*VECs damaged by the parasites against that completely lysed with the Lysis buffer. To directly monitor the cytolytic events, the *h*VECs monolayer damaged by parasites was visualized with IFA and the lysed area was measured, as shown in Fig. 8B.

For the drug treatment control, we separately pretreated the parasites with Edelfosine, BAPTA-AM, A23187, and Latrunculin B in TYI medium for 90 min on rotation before analysis. After washing with the medium to remove extracellular drugs, the trophozoites were immediately inoculated into *h*VECs culture for assays (lines 573). Thus, the *h*VECs cytotoxicity may not be from the direct effects of drugs.

For the presence of PIC in the co-culture system, we supplied the effect of PIC treatment on *h*VECs viability in S12 Fig. D and described in line 412.

12. Finally, I recognize that the authors are not native English speakers, and I hesitate to say so because I appreciate the authors' efforts to produce an article in a language other than their own, but the material is difficult to read and the grammar needs to be improved. The authors conducted a thorough work, and enhancing the English will make the paper much more enjoyable to read.

Re: We appreciate the reviewer’s comment and the manuscript has been edited to improve readability.

Reviewer #2: No new experiments or modifications are required.

Reviewer #3:

The cell localization of TvPI4P5K is not convincing. Fig. 2E, TvPI4P5K in TH17 is

possibly on the membrane, however, why did the authors select just the displayed cell with a very strong signal only on one site? How many cells displayed this pattern?

Re: For data consistency, we replaced the IFA image of Fig. 2E with a typical one to show the partial plasma membrane localization of *Tv*PI4P5K. Also, a density gradient ultracentrifugation coupled with western blotting demonstrated that the *Tv*PI4P5K signal was enriched in the plasma membrane fractions and various membrane compartments, supporting our observation on *Tv*PI4P5K plasma membrane localization (S3 Fig. C, line 169, line 762).

In Fig. 3D, TvPI4P5K is mainly in the cytosol. PCC supported some colocalization with PIP2, however, this is not because of colocalization on the membrane, but also colocalization in the cytosol.

Re: We appreciate the reviewer's suggestion. When the *Tv*PI4P5K signal was partially distributed in the cytoplasm and particular plasma membrane regions but PIP_2 mainly in the plasma membrane, we used the signal intensity distribution on a line to highlight the plasma membrane *Tv*PI4P5K signal partially superimposed with PIP_2. However, this partial plasma membrane colocalization might be counteracted when evaluating the whole-cell signals PCC.

In this revision, we removed the whole-cell PCC from the manuscript and emphasized the partial plasma membrane localization of the *Tv*PI4P5K signal, which may be associated with PIP_2 production around the plasma membrane. In addition, the signal distribution in the IFA image was visualized by the 2.5D view function of Zen software (Zeiss), by which the intensities in two-dimensional image were converted into a height map and represented by the extension in the Z-direction, indicating the partial cell membrane residence of *Tv*PI4P5K, which colocalized with the PIP_2 signals in particular regions. Data are supplied in Figures S3, S4, and S10.

Fig 3E. The effect of Edelfosine, the membrane localization of TvPI4P5K is not convincing. The yellow line is drawn in the area with membrane localization but there is a significant cytosolic signal in the other part of the cell. In general, for colocalization, it is preferable to use 2D histogram for channels to cover the whole cell rather than a line (yellow lines in Figures) as the result depends on the line position. The authors should provide more convincing IF figures for TvPI4P5K and if possible westerns of cell fractions.

Re: We appreciate the reviewer's comment. It is preferable to use 2D histogram and scatter plot to evaluate the extent of colocalization for the whole-cell signal. However, the PCC or 2D histogram for whole-cell signals might omit the partial colocalization signals. *Tv*PI4P5K signal was detected in the cytoplasm and partially concentrated in

specific cell membrane regions. To provide more convincing evidence, we directly visualized the colocalization in the images of the 2.5D view image to show the plasma membrane *Tv*PI4P5K signals partially colocalized with PIP_2 (S4 Fig.). Meanwhile, the whole-cell colocalization PCC value was removed from this revision.

Alternatively, density gradient ultracentrifugation coupled with western blotting was included to demonstrate that the *Tv*PI4P5K signal was enriched in the plasma membrane fractions and uncertain membrane compartments, supporting our observation of *Tv*PI4P5K plasma membrane localization (S3 Fig. C, line 169, line 762).

396. The authors used LDH assay to monitor the cytotoxicity of trichomonads on hVECs. However, this test is not suitable for T. vaginalis as there is a strong LDH activity in the parasite cytosol. It is impossible to distinguish the origin of LDH activity upon coincubation of T. vaginalis with hVECs. Moreover, if the atmosphere contains oxygen during the coincubation, the dying of trichomonad is significant. This experiment should be performed using more specific detection such as radiolabelling of target cells.
Re: We want to express our most profound apology. Since radiolabeling materials are restricted in our institute, we attempted to explain the methodology and supply supportive information for the LDH cytotoxicity assay. First, a trypan blue exclusion viability assay was included to demonstrate that the parasite did not die significantly until 4 hr of culture in 5% CO_2 (S12 Fig. A, line 402). Based on this observation, our assay focused on the early 1-hr cytotoxic events. Second, the spent medium from the co-culture system was centrifuged to remove parasites and *h*VECs before the LDH activity assay (line 748). Third, the spent medium from the parasites culture without *h*VECs was collected for an LDH activity assay which detected low LDH activity (S12 Fig. C, line 411). We supplied the above supportive data to exclude the LDH activity leakage from the parasites in the cytotoxicity assay.

219. The conditions of the cytoadherence test are not provided in sufficient detail to evaluate its relevance. Which medium was used for coincubation and under which atmosphere it was performed? This is critical information as trichomonads are sensitive to oxygen and incubation under aerobic conditions will induce oxygen-dependent changes in trichomonad physiology and apoptosis which compromises these results.
Re: The cytoadherence assay was performed in keratinocyte serum-free medium under 5% CO_2. Since the trichomonad did not die significantly until 4 hours of culture under this test condition (S12 Fig. A, line 402), we monitored *T. vaginalis* cytoadherence within 1 hour. The condition is stated in the materials and methods of the revised

manuscript (line 722) and the related reference (28).

412 and 698. Cytoadherence study. Similarly as above. In which atmosphere trichomonads and hVEC cells were incubated in a minimal thin layer of culture media for 1 hr? If the atmosphere was aerobic, they were dying during the incubation and the experiments provide no relevant information.
Re: The cytotoxicity, cytolysis, and cytoadherence assay were performed in keratinocyte serum-free medium in 5% CO_2. Since the Trichomonad starts to die until 5 hours of culture under this test condition (S12 Fig. A, line 402), we focused on the early 1-hour events to rule out the influence of the parasites. The conditions are detailed in the materials and methods of the revised manuscript (lines 723, 737, and 748). A similar protocol for the *T. vaginalis* cytotoxicity assay had also been reported in the reference study (11).

Part III – Minor Issues: Editorial and Data Presentation Modifications
Please use this section for editorial suggestions as well as relatively minor modifications of existing data that would enhance clarity.

Reviewer #1: Minor comments:

- Page 13, line 167: ¨The data and was consistent with the earlier RT-PCR (Fig. 1F), supporting the potential correlation of TvPI4P5K and PIP2 expression with T. vaginalis cytoadherence¨. It should be Fig 1E
Re: It was corrected to Fig. 1D (line 159).

- Replace ¨nonadherent isolates¨ by ¨less adherent isolates.
Re: "Nonadherent isolates" were replaced by "less adherent isolates" (lines 26, 106, 113, 122, 124, 125, 158, 500, 502, 508, 1131).

- Page 20, line 271. Introduce that BAPTA-AM is a cell-permeant chelator that can be used to control the level of intracellular Ca 2+.
Re: The introduction was stated in line 268 of the revised manuscript.

- Explain what CG is in the figure legend 4
Re: Calcium Green (CG) is explained in the legend of Fig. 4 (line 280).

- Page 24, line 312: explain that ¨A23187¨ is a Calcium Ionophore
Re: It was described in line 313 of the revised manuscript.

Reviewer #2: There are few minor issues that need to be considered to improve clarity, such as:

1. In Fig. 1A please explain what are describing the 2nd part of the figure in which the signal intensisty is so weak. Are they representing negative controls? Please include the missing information in the corresponding figure legend.

Re: Detection by anti-PIP2 (upper panel) and anti-PIP3 (lower panel) antibodies has been added in the legend of Fig. 1A (line 140).

2. P7, line 167. Please chech this sentence. It appears incomplete.

Re: It was corrected (line 158).

3. P7, line 184. …His-TvPI5P5K… It should be His-TvPI4P5K? Please correct it as need it.

Re: This has been corrected (line 178).

4. P17, lines 267, 269. …GC… It should be CG (Calcium green). Please correct them as needed.

Re: This has been corrected. (line 263)

5. Line 487 and S6 line 66. …TvPI5P5K…It should be TvPI4P5K?

Re: This has been corrected (line 499).

Reviewer #3: Fig 2. Why did authors coIP HA-tagged TvPI4PK? Is the expression from the vector low that the protein cannot be detected directly on the western blot of cell lysate or cell fractions? Based on HA tag signal in IFA, it should be detectable on westerns.

Re: The western blotting procedure was modified to detect endogenous *Tv*PI4P5K or recombinant HA-*Tv*PI4P5K signals in the parasite total lysates (Figs. 2C and 2G).

Fig 7. The authors showed that A23187 reversed several effects of iron depletion, including calcium content, transformation to amoeboid forms, cytotoxicity etc. This is a very interesting result, but it should be discussed in more detail with available publications reporting the effect of iron on trichomonad gene expression and physiology. The discussion line 514 and following "Iron and signal transduction" is very limited.

Re: As per the reviewer's comment, the effect of iron on trichomonad gene expression and physiology was discussed in line 528.

Regarding the host-parasite interactions, extracellular calcium may influence trichomonad recognition and binding fibronectin (54) and enhance *Tv*CLP-mediated *T. vaginalis* aggregation on the host cells (4). Our study further expands the role of calcium to function as an intracellular second messenger contributing to *T. vaginalis* pathogenesis.

Iron is an essential nutrient for *T. vaginalis* and is acquired via a highly specific receptor-mediated mechanism from the host (55). The absence of iron in the culture medium decreases cell growth and triggers morphological changes from the amoeboid to a rounded flagellate form by an unknown mechanism. Iron exerts versatile roles in parasite growth and virulence, including the expression of genes required for metabolism (19) or virulence (21, 22). Also, iron increases *T. vaginalis* resistance to complement lysis for evading host immunity (23). Iron may modulate histone acetylation at H3K27 and tri-methylation at H3K4 within the coding region of iron-responsive *ap65-1* and *pfo* genes, epigenetically regulating cytoadherence (16). Meanwhile, iron promotes cAMP-dependent *Tv*PKAc activation to phosphorylate the Myb3 transcription factor for rapid nuclear import required for regulating *ap65-1* gene expression (17).

line 219. The author should specify in the text that trichomonads were coincubated with hVEC cells to test the cytoadherence.
Re: The description was stated in line 210 of the revised manuscript.

Fig 1. D. The alignment is more suitable for supplementary materials.
Re: The sequence alignment was moved to S2 Fig.

Material and methods.
line 545. The authors should specify how the T. vaginalis strains were cryopreserved and to which publicly available culture collection were deposited.
Re: For *T. vaginalis* cryopreservation, 2× 10^7 trophozoites of the clinical isolates were suspended in 1 ml of TYI medium with 7.5% DMSO and kept in the freezing container filled with isopropanol and then placed in a -80°C freezer over 24 hr. All isolates were deposited in the biosafety chamber of our institute for research only (National Taiwan University, College of Medicine). The procedure for cryopreservation was described in the materials and methods of the revised manuscript (line 581).

563. The plasmid must be clearly described. Reference 60 (line 568) provides no information but another reference for the article in which the plasmid is not described

in detail either. It is important to provide information on the FLP promotor, how strong the gene expression is upon this promotor etc.

Re: The promoter region (-786 to +11) of the Fibronectin-like protein-1 (FLP-1) gene is used in our plasmids to overexpress the target genes at levels 5- to 8-fold higher than the endogenous genes for various assays (lines 165, 188, and 595).

624. The authors used a confocal microscope, however, there is no information on how many sections were performed, their size, and how many sections were used for the figure construction.

Re: The fluorescence images were captured by confocal microscopy (Objective: Plan-Apochromat 100/1.40 Oil Ph3, LSM-780) in a single Z-slice as described in the materials and methods (line 657).

698. The CFSE labeling needs a description.

Re: The trophozoites were incubated with 5 μM CFSE in PBS with 1% BSA on a gentle rotation at 37°C for 30 min. After washing three times with PBS, the parasites were suspended in medium for analysis (line 719).

706. Cytolysis. I miss the information about the atmosphere and medium which was used for co-incubation.

Re: The cytolysis and cytotoxicity assays were performed in keratinocyte serum-free medium under the atmosphere with 5% CO_2 as detailed in the materials and methods of the revised manuscript (lines 730 and 748).

Fig E, I. The "merge" of IF with DIC is superfluous. The same for double merge figures with and without lines indicating sections for signal intensity distribution. A single merge with lines would be sufficient, but 2D histogram instead of lines would be more informative.

Re: The images merged IF with DIC were removed from this revised manuscript. Our current findings attempt to emphasize that partial, but not all, *Tv*PI4P5K is located at the plasma membrane colocalizing with PIP_2 in particular regions (line 163). Although the 2D histogram is preferable for presenting whole-cell overall signal colocalization, it may overlook those signals partially colocalized in certain areas. To highlight the partially colocalized *Tv*PI4P5K and PIP_2 signals at the plasma membrane, we have removed the whole-cell colocalization PCC and supplied the 2.5D view images in the revised manuscript to exhibit the plasma membrane colocalization of *Tv*PI4P5K and PIP2 at specific regions (S3, S4, S10 Figs.). It may be more suitable for Figs. 2E and

2I interpretation.

271 Please specify for readers in the text that BAPTA-AM is a calcium chelator.
Re: "BAPTA-AM is an intracellular calcium chelator" was specified in line 268 of the revised manuscript.

369. Please specify in the text what is A23187 - calcium ionophore
Re: "A23187 is a calcium ionophore" was specified in line 313 of the revised manuscript.

196. Please, spell out FLP.
Re: Fibronectin-like protein (FLP) was stated in the revised manuscript (lines 188 and 595).

400. Please, sell out MOI.
Re: Multiplicity of infection (MOI) was stated in the corresponding context of the revised manuscript (line 401).

The authors should be more careful with the interpretation of the effect of K136A mutation as an effect of PIP2 signaling: subtitle 303, 329, 396, and the title.
Re: The effect of *Tv*PI4P5K K136A mutation on depletion of PIP_2 in PIP_2 signaling was mentioned in line 165, as well as lines 264, 310, 406, and 422.

PLOS authors have the option to publish the peer review history of their article (what does this mean?). If published, this will include your full peer review and any attached files.

If you choose "no", your identity will remain anonymous but your review may still be made public.

Do you want your identity to be public for this peer review? For information about this choice, including consent withdrawal, please see our Privacy Policy.
Reviewer #1: No
Reviewer #2: No
Reviewer #3: No

Figure Files:

While revising your submission, please upload your figure files to the Preflight Analysis and Conversion Engine (PACE) digital diagnostic tool, https://pacev2.apexcovantage.com. PACE helps ensure that figures meet PLOS requirements. To use PACE, you must first register as a user. Then, login and navigate to the UPLOAD tab, where you will find detailed instructions on how to use the tool. If you encounter any issues or have any questions when using PACE, please email us at figures@plos.org.

Data Requirements:

Please note that, as a condition of publication, PLOS' data policy requires that you make available all data used to draw the conclusions outlined in your manuscript. Data must be deposited in an appropriate repository, included within the body of the manuscript, or uploaded as supporting information. This includes all numerical values that were used to generate graphs, histograms etc.. For an example see here on PLOS Biology: http://www.plosbiology.org/article/info%3Adoi%2F10.1371%2Fjournal.pbio.1001908#s5.

Reproducibility:

To enhance the reproducibility of your results, we recommend that you deposit your laboratory protocols in protocols.io, where a protocol can be assigned its own identifier (DOI) such that it can be cited independently in the future. Additionally, PLOS ONE offers an option to publish peer-reviewed clinical study protocols. Read more information on sharing protocols at https://plos.org/protocols?utm_medium=editorial-email&utm_source=authorletters&utm_campaign=protocols

---

In compliance with data protection regulations, you may request that we remove your personal registration details at any time. (Remove my information/details). Please contact the publication office if you have any questions.
